# PSMA1, PSMA5, and PSMB2 serve as prognostic biomarkers and are correlated with tumor-infiltrating leukocytes in HCC

**DOI:** 10.1080/15592294.2026.2726624

**Published:** 2026-09-14

**Authors:** Baitul Islam, Weiye Xu, Zaid Ur Rehman, Ru Zeng, Thekra Khushafa, Haiyang Yu, Jufang Huang

**Affiliations:** a Department of Anatomy and Neurobiology, School of Basic Medical Sciences, Central South University, Changsha, China; b Molecular Biology Research Center and Hunan Province Key Laboratory of Basic and Applied Hematology, School of Life Sciences, Central South University, Changsha, China

**Keywords:** Proteasome, biomarkers, prognosis, HCC, cancer

## Abstract

Hepatocellular carcinoma (HCC) ranks as the third leading cause of cancer-related death. Proteasome (PSM) is the main intracellular proteolytic system in higher eukaryotic cells. It has been reported to be involved in the tumor’s onset, metabolism, and survival, and it has been recognized as a therapeutic target for many human cancers. The 20S proteasome subunits, specifically proteasome 20S subunit alpha 1 (PSMA1), proteasome 20S subunit alpha 5 (PSMA5), and proteasome 20S subunit beta 2 (PSMB2), are overexpressed in various malignancies, including HCC. Nevertheless, the exact role of these genes in HCC prognosis remains only partially understood. Moreover, a reliable predictive biomarker for HCC is essential for supporting the implementation of personalized therapies. Therefore, this study employed a comprehensive bioinformatics approach, integrating data from cBioPortal, Human Protein Atlas (HPA), Oncomine, STRING Viruses, Kaplan–Meier plotter, and other established high-throughput databases and tools. The current study thoroughly examined the expression levels, methylation, and genomic alterations of PSM and their correlation with tumor-infiltrating leukocytes. PSMA1, PSMA5, and PSMB2 are overexpressed in various cancers, including HCC, and their expression levels are associated with tumor grade and stage. The abundance of these genes is linked to diminished DNA methylation levels and genome alterations. Moreover, there was a significant association between the expression levels of these genes and poor prognosis and immune cell infiltration. In conclusion, this study proposes PSMA1, PSMA5, and PSMB2 as biomarkers for HCC.

## Introduction

Hepatocellular carcinoma (HCC) is the sixth most common cancer around the globe and the third leading cause of cancer-related mortality [1]. Various risk factors contribute to the initiation of HCC, with approximately 80% of cases associated with viral infections [2]. Chronic infections by Hepatitis B virus (HBV) and Hepatitis C virus (HCV), play a significant role in HCC pathogenesis [3]. Notably, HCV is responsible for 21% of virus-associated HCC cases [4]. However, the intricate mechanisms underlying this association are complex and not fully elucidated [5]. HCV, in particular, has garnered attention for its significant role in liver diseases, with an estimated 1.7 million new infections annually and a staggering 71 million individuals afflicted globally [6]. Left untreated, HCV can establish viral persistence, leading to liver cirrhosis in approximately 20% of patients, with a subsequent annual HCC incidence of 1–4% [7]. While direct antiviral agents (DAAs) show promise in eradicating HCV, the risk of HCC development in patients with advanced liver disease remains a concern. Recent reports indicate an unexpected increase in HCC incidence post-DAA treatment, underscoring the incomplete understanding of HCV’s oncogenic mechanisms and the persistence of cancer risk even after viral clearance. Despite advancements, conventional diagnostic methods often fall short in predicting or detecting HCC at an early stage. Thus, identifying biomarkers specific to HCC holds promise to enable timely prediction and early detection, thereby guiding targeted therapeutic interventions.

The proteasome constitutes the principal intracellular proteolytic system within higher eukaryotic cells, localized in both the nucleus and cytoplasm. Its role in protein degradation is crucial for orchestrating physiological stress responses and signal transduction by regulating protein abundance and eliminating misfolded and damaged proteins. Additionally, proteasome facilitates the maturation of certain polypeptide precursors into biologically active molecules [8,9]. Comprising the 20S core particle and the 19S regulator, the 26S proteasome represents a sizable multiprotein complex. Within the 20S core complex, consisting of seven distinct subunits, each of PSMA1-7 and PSMB1-7, PSMAs serve as integral constituents essential for proteasome assembly and binding to the 19S regulatory complex [10]. Notably, dysregulation of PSMAs has been observed in various cancers, including breast, bladder, colorectal, gastric, HCC, head, and lung cancer, where these subunits are frequently upregulated relative to normal tissues [11,12]. The β subunits encoded by the PSMBs gene family are responsible for post-ubiquitination proteasomal degradation of aberrantly folded or short-lived intracellular proteins. The ubiquitin-proteasome system (UPS), mediated by PSMBs, degrades over 80% of intracellular proteins [13]. Dysregulation of UPS has been implicated in various cancers, contributing to aberrant proliferation, inhibition of apoptosis, and degradation of tumor‑suppressive proteins, thereby impeding the growth and division of cancer cells [14].

Even though all proteasome subunits contribute to the overall structure and functions of 26S proteasome but also accumulating evidence shows that individual subunits possess specific biological roles beyond maintaining proteasome architecture. PSMA1 and PSMA5 are α-ring subunits which control substrate entry into the catalytic chamber, and which are required for correct proteasome assembly/stability. Changes in these α subunits may therefore affect proteasome function, protein homeostasis and signaling pathways contributing to tumor progression [11,12]. Conversely, PSMB2 is a β-ring structural subunit aiding in the architecture and functional cohesion of the catalytic core, thereby implicating proteasome-directed protein degradation that regulate progress through the cell cycle, apoptosis and oncogenic signaling [14]. In contrast to catalytic β subunits such as PSMB5, which have been studied extensively as therapeutic targets, the biological and clinical implications of non-catalytic subunits PSMA1, PSMA5 and PSMB2 remain largely unknown especially with respect to HCC tissues [11,12]. This led to the hypothesis that we could systematically characterize their prognostic significance and possible biological function in HCC.

Although various proteasome subunit genes have been associated with tumor initiation, not all are equal. Catalytic subunits β (β1–7] have received considerable attention due to their relevance as targets for FDA-approved proteasome inhibitors; however, the clinical importance of structural subunits including PSMA1, PSMA5, and PSMB2 is not well understood [15]. Recent reports indicate that these genes play roles in proteasome assembly, substrate gating, and preservation of the proteasome complex, which can, therefore, indirectly affect cell division and survival as well as oncogenic signaling. Yet their importance as a prognostic significance for HCC has yet to be evaluated. Thus, this study was designed to assess the expression signatures, potential prognostic value, genomic alterations, co-expression pattern, protein interaction network, and immune correlations of PSMA1, PSMA5, and PSMB2 using a bioinformatic approach.

**Figure 1.**
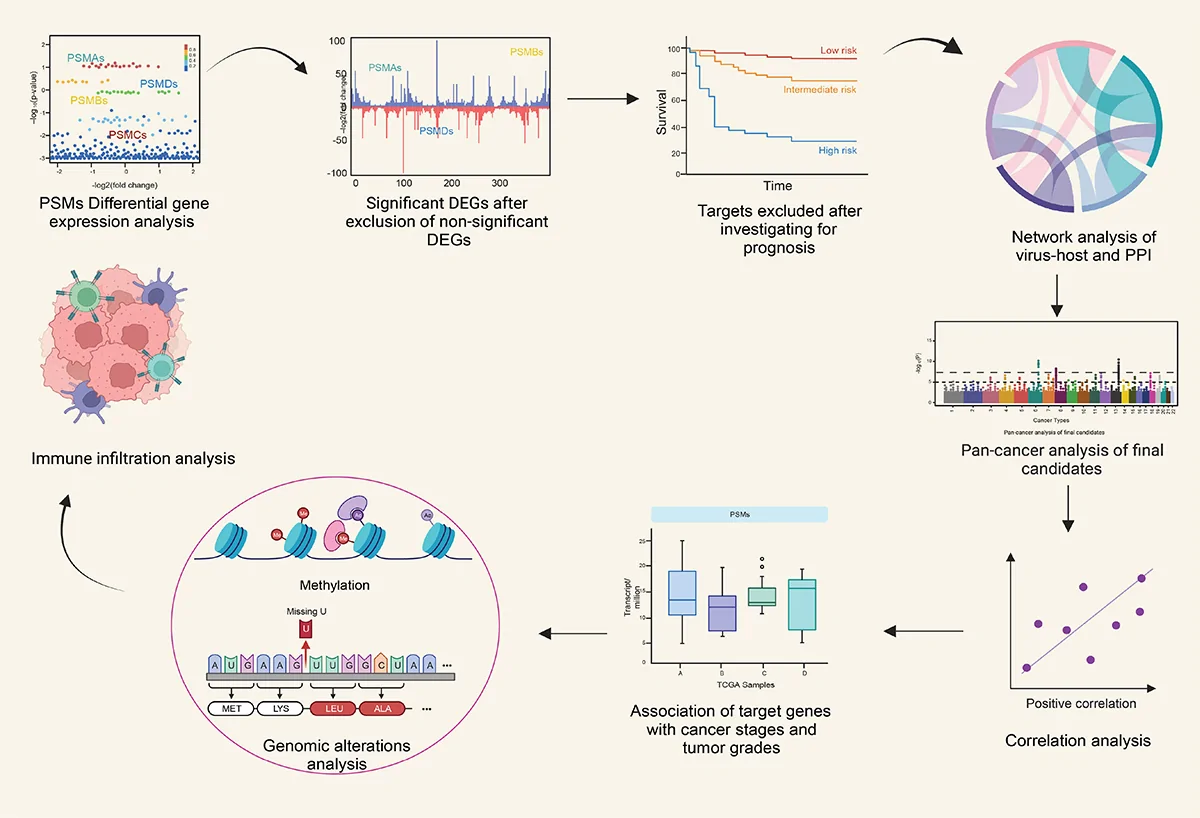
Workflow for the current study.

Figure 1 delineates the comprehensive and innovative methodological framework employed in this study. These findings hold promise for the future of cancer research and therapeutic development.

## Materials and methods

### Description of the data and datasets

All data used in the current study are available online and were obtained from different databases, including Gene Expression Omnibus (GEO), HPA, and The Cancer Genome Atlas (TCGA).

### Analysis of differential expressions of PSMA1, PSMA5, and PSMB2 in pan-cancer and HCC

Differential expressions of PSMA1, PSMA5, and PSMB2 were evaluated across various cancers, including HCC, using TIMER 2.01. TIMER (https://cistrome.shinyapps.io/timer/, accessed in January 2025) is a valuable, comprehensive online database that provides numerous analytical functions across diverse cancers [16]. TIMER contains 10,897 samples from various cancer types in the TCGA data. TIMER was used to evaluate PSMA1, PSMA5, and PSMB2 expression levels in pan-cancer. We analyzed the PSMA1, PSMA5, and PSMB2 expression levels in 33 cancers. Additionally, the expression levels of PSMs were analyzed in HCC tumors (370 samples) versus normal liver tissues (50 samples) using UALCAN (http://ualcan.path.uab.edu/analysis.html, accessed in January 2023). UALCAN (The University of Alabama at Birmingham cancer data analysis) is a user-friendly, interactive web resource that collects RNA sequencing and patient data for different cancer types from the TCGA database. UALCAN allows users to evaluate the expression levels of genes with numerous clinical factors, including pathological grades and cancer stages, and generates high-quality graphics using cancer omics data. We also used the database for in-depth analysis of PSMA1, PSMA5, and PSMB2 expression across subgroups defined by pathological grade and tumor stage. Furthermore, this study confirmed the overexpression of PSMA1, PSMA5, and PSMB2 in HCC using HCCDB (http://lifeome.net/database/hccdb/home.html, accessed in February 2025). HCCDB is a user-friendly one-step online source for HCC gene expressions, containing 3917 clinical sample data from TCGA and Genotype-tissue Expression (GTEx). Including 13 microarray datasets from GEO and 2 RNA-seq datasets from TCGA and the International Cancer Genome Consortium (ICGC). This study used HCCDB to analyze and visualize the differential expression of PSMA1, PSMA5, and PSMB2 in HCC tumors versus cirrhosis and normal tissues in 12 datasets.

### Analysis of protein expression in human clinical samples

This study assessed the immunohistochemistry analysis for PSMA1, PSMA5, and PSMB2 within the HPA database to compare the protein expression statuses of our target genes in tumor and normal liver tissue samples. HPA (www.proteinatlas.org, accessed in February 2025) is an online database providing information on expression sequences, pathologies, and distributions in various cancers. Based on the intensity of the staining, the protein expression levels were classified as ‘high,’ ‘medium,’ ‘low,’ and ‘not detected.’

### Survival analysis of HCC patients

To evaluate the transcriptomic effects of PSMA1, PSMA5, and PSMB2 on HCC patients, the KM-curves were generated using the GEPIA, Kaplan–Meier plotter (KM) database, and UALCAN to assess the overall survival (OS).

### Expression profile and correlation analysis

LinkedOmics (http://www.linkedomics.org/login.php, accessed in February 2025) constitutes a web-centric repository housing HCC data derived from 32 discrete TCGA datasets. Within the confines of this study, the TCGA LIHC dataset underwent scrutiny to identify genes exhibiting positive co-expression with PSMA1, PSMA5, and PSMB2, facilitated by the resources provided by the LinkedOmics database. The foremost genes manifesting positive co-expression were subsequently extracted and visualized through the depiction of heat maps. Furthermore, the correlation between PSMA1, PSMA5, and PSMB2 was conducted using GEPIA (http://gepia.cancer-pku.cn, accessed in February 2025).

### Functional enrichment and protein–protein Interaction (PPI) analysis

The current study used DAVID to analyze Gene ontology (GO) and Kyoto Encyclopedia of Genes and Genomes (KEGG) pathway enrichment for the co-expressed genes. The top 50 positively correlated significant genes with PSMA1, PSMA5, and PSMB2 were introduced to the Database for Annotation, Visualization, and Integrated Discovery (DAVID). DAVID (https://david.ncifcrf.gov/, accessed in February 2025) is an online database for functional annotation and pathway enrichment analysis. The GO terms and KEGG pathways results were downloaded and visualized using the ggplot2 package of R software. Additionally, the virus-host protein interaction network was constructed using STRING viruses. STRING viruses is an online database containing 2031 organisms with 9,643,763 proteins. STRING viruses construct known and predicted protein–protein interactions between viruses or their proteins and target human genes. In the current study, we introduced our target human genes (PSMA1, PSMA5, and PSMB2) to the STRING viruses (http://viruses.string-db.org/, accessed in February 2025) database. The parameters for establishing the virus-host PPI with string viruses were defined as follows: Hepatitis C virus genotype 1a and Homo sapiens were chosen as the viral and host components, respectively, from a predetermined selection menu. A maximum of 20 interactions within each primary and secondary shell was specified, while the minimum interaction score required was set to a high confidence level (0.7). Subsequently, the pertinent data were acquired and subjected to network analysis and visualization using Cytoscape 3.9.1 software. To further understand the physical and functional association among our target genes and their co-targeted genes by HCV, the top 10 genes (based on the degree of centrality) were selected and introduced to the geneMANIA database for subsequent analysis. The GeneMania platform (http://genemania.org/, accessed in February 2025) was used to conduct gene co-expression, network, and functional enrichment analyses of the top 10 HCV target genes.

### Genomic alterations, methylation, and copy number alterations analyses

The methylation levels of PSMA1, PSMA5, and PSMB2 were examined using UALCAN. To elucidate the correlation between the methylation status and mRNA expression levels of these genes, c-Bioportal was employed. c-Bioportal (https://www.cbioportal.org, accessed in February 2025), an online repository for comprehensive analysis of cancer omics data. Specifically, a dataset encompassing 360 liver hepatocellular carcinoma (LIHC) samples sourced from TCGA was selected for methylation analysis. Alongside methylation analysis, genomic alterations pertaining to the target genes were assessed in HCC. Moreover, the relationship between PSMA1, PSMA5, and PSMB2 and copy number alterations (CNAs) was investigated utilizing c-Bioportal.

### Molecular docking

The PDB structures of human receptors and viral proteins were retrieved from RCSB PDB (Structural Bioinformatics Protein Data Bank) in PDB format. The RCSB PDB IDs of the respective proteins are given in Supplementary Table S1. The structures of viral proteins and human receptors were visualized and processed (e.g., removal of HETATOMS) using UCSF Chimera. The three human receptors named PSMA1 (F chain), PSMA5 (E chain), and PSMB2 (K chain) were docked against eight viral proteins to ascertain the molecular interaction between human receptors and viral proteins. The molecular docking was performed using Cluspro, an online web server that performs rigid-body docking by sampling billions of molecular conformations. The docking algorithm uses PIPER, a program based on the Fast Fourier Transform (FFT), to compute the interaction energy (E) between two proteins. The interaction energy E is calculated by combining different energies, as shown in Eq 1.

(1) $$E=0.40E_{\mathrm{rep}}+-0.40E_{\mathrm{att}}+600E_{\mathrm{elec}}+1.00E_{\mathrm{DARS}}$$

Where $E_{\mathrm{att}}$ and $E_{\mathrm{rep}}$ is the attractive and repulsive contribution to the van der Waals interaction energy, $E_{\mathrm{elec}}$ denotes the electrostatic energy. $\;E_{\mathrm{DARS}}$ denotes the pairwise structure-based potential constructed by the ‘decoys as the reference state’ (DARS). After rigid-body docking, the clustering algorithm based on RMSD was used to cluster the generated structures. From billions of conformations, 1000 lowest energy structures were generated. Lastly, the structures from the clustering steps were energy minimized, and the most stable and energetically favorable complexes were generated. Cluspro produced 10 stable complexes; the one with the lowest energy was selected from this set. The most stable complex of each viral protein against human receptor was selected and visualized in the USCF chimera. The selected complexes were subjected to molecular interaction analysis using LigPlot^+^. LigPlot^+^ is a visual interface that creates schematic diagrams of interactions between proteins and ligands. These diagrams include different kinds of interactions, such as hydrogen bonds, Hydrophobic contacts, and pi–pi stacking interactions. Hydrogen bonds (non-covalent interactions between hydrogen and electronegative atoms) are important in determining the precise binding orientation and specificity between interacting proteins. Hydrophobic contacts occur between nonpolar molecules and have a significant role in stabilizing PPIs. These interactions play an important role in the stability, specificity, and functionality of the complexes formed.

### Cell transfection

HepG2 cells were plated in 6-well plates at a density of 1 × 10^5^ cells per well and allowed to adhere overnight. The pCMV3-C-FLAG was used to express Hepatitis C virus (HCV-1b) Core (HCV-Core). The cells were then transfected using Lipofectamine 3000. Once the cells reached 70–75% confluence, the cell medium was replaced with Opti-MEM containing the relevant plasmids and the transfection reagent. The mixture was incubated for 20 min before being added to the cells. The complete medium was restored after 6 h, and cells were harvested 48 h after transfection. HCV-Core expression efficiency was verified by qPCR and WB after 48 h.

### Western blotting (WB)

WB was performed as previously described [17]. In summary, the proteins were extracted through Radioimmunoprecipitation assay (RIPA) lysis buffer (CoWin Biosciences, Jiangsu, China). The extracted proteins were measured using a BCA protein assay kit (CoWin Biosciences, Jiangsu, China) and loaded as 20–30 mg per well. The protein samples were separated with 12% sodium dodecyl sulfate-polyacrylamide (SDS) gel electrophoresis and transferred into a solid nitrocellulose membrane. The blot membranes were then incubated with the primary antibodies overnight after they were blocked with a blocking solution (5% non-fat dry milk) for 90 min. Afterward, the membranes were washed with TBST and incubated with the secondary antibodies. Next day, the membranes were incubated with secondary antibodies after washing them three times with TBST. The bands were visualized with chemiluminescence and were measured with ImageJ. The antibodies used for WB were supplied by AntGene (Wuhan AntGene Biotechnology).

### Immune cell infiltration analysis

TIMER 2.0 provides a comprehensive analysis of immune estimation by applying a deconvolutional statistical method. By utilizing the immune estimation module, TIMER facilitated the correlation analysis between the expression profiles of designated genes and the infiltration patterns of diverse immune cell populations, encompassing B cells, CD4+ T cells, CD8+ T cells, dendritic cells, macrophages, and neutrophils. Moreover, the TIMER correlation module enabled the exploration of the relationship between PSMA1, PSMA5, and PSMB2 and immune markers. Furthermore, TIMER analyzed the correlation between the top 10 target genes of HCV.

### Statistical analysis

The web tools and data platforms employed in the current investigation computed adequate statistical analyses. The Spearmen correlation was used to assess the correlation between PSMA1, PSMA5, PSMB2, and other gene markers in the TIMER database and the correlation between co-expressed genes in the linkedOmics database. Using the c-Bioportal database, the Spearman correlation was utilized to assess the relationship between co-expressed genes. A *p*-value of 0.05 was considered statistically significant. Statistical significance is indicated as: *p < 0.05, **p < 0.01, ***p < 0.001, ****p < 0.0001, ns: not significant.

## Results

### Expression levels of PSMA1, PSMA5, and PSMB2 in pan-cancer and in-depth validation in HCC

**Figure 2.**
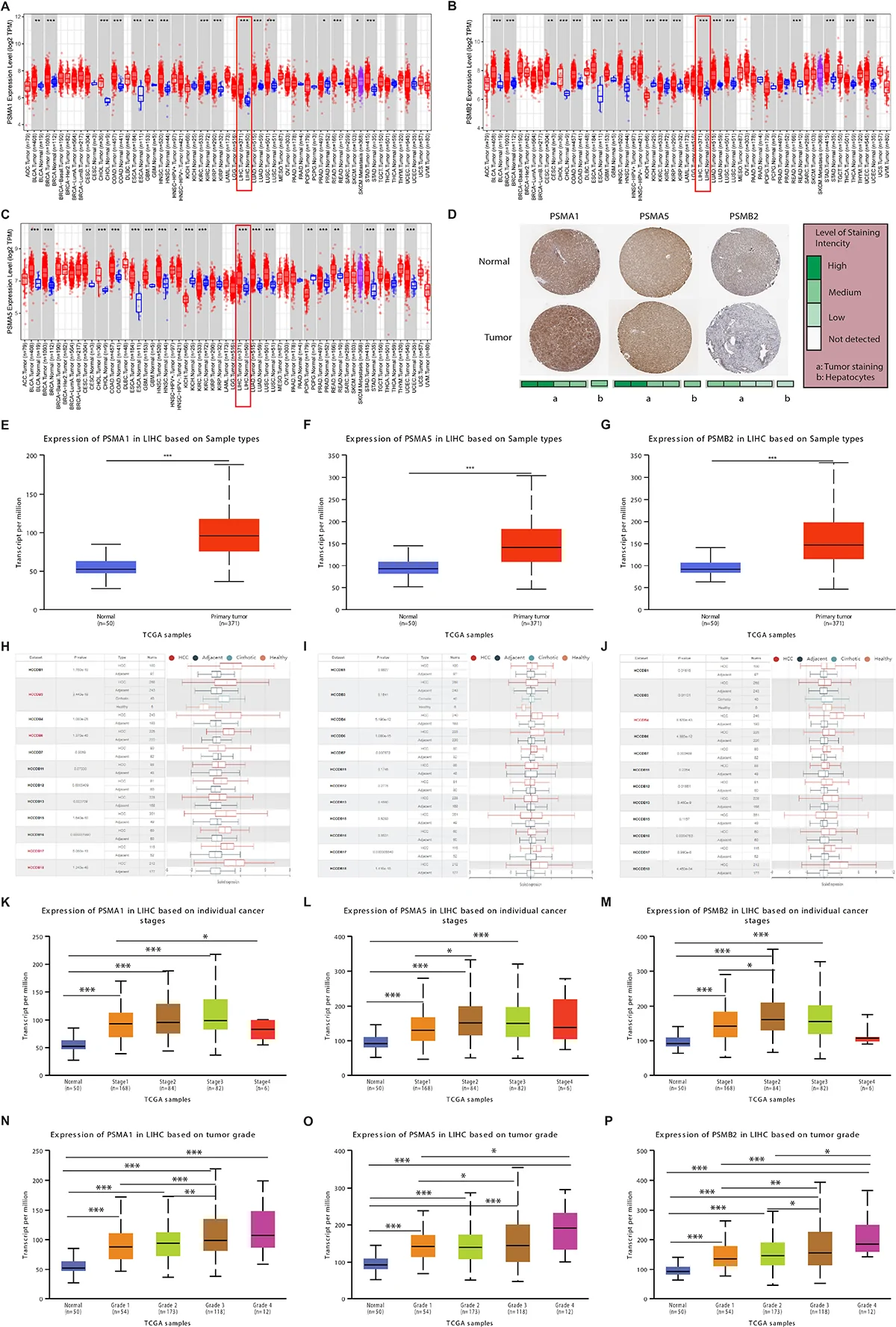
The PSMA1, PSMA5, and PSMB2 expression levels in various cancer types. (A-C) PSMA1, PSMA5, and PSMB2 mRNA expression levels in various tumor types, as determined by TCGA data, are shown in the TIMER database. (D) PSMA1, PSMA5, and PSMB2 immunohistochemistry pictures from the HPA of HCC tumor and normal tissues. (E-G) box plots in UALCAN display the PSMA1, PSMA5, and PSMB2 mRNA expression levels in HCC tumors and normal tissues. (H-J) plots showing the expression of PSMA1, PSMA5, and PSMB2 in HCC from HCCDB. (K-M) association between PSMA1, PSMA5, and PSMB2 expression levels in normal tissues and cancer stages. (N–P) PSMA1, PSMA5, and PSMB2 expression levels in normal tissues and different grades of HCC tissues. Statistical significance is indicated as: *p < 0.05, **p < 0.01, ***p < 0.001, ****p < 0.0001.

TIMER is a user-friendly interactive web tool for analyzing multidimensional cancer omics data. We obtained the mRNA expression levels of PSMA1, PSMA5, and PSMB2 in numerous cancer types, including cholangiocarcinoma, lung adenocarcinoma, and hepatocellular carcinoma. All three genes were significantly overexpressed in tumors compared to the normal tissues (Figure 2(A–C)). The differential expression of our target genes was then evaluated in HCC, particularly within the UALCAN database. As presented in Figure 2(E–G), PSMA1, PSMA5, and PSMB2 were significantly overexpressed in HCC compared to the normal liver tissues. By observing the immunohistochemistry results from HPA, protein expression levels of PSMA1, PSMA5, and PSMB2 were consistently higher in Hepatocellular carcinoma than in normal liver tissue samples. As shown in Figure 2(D), ‘medium’ to ‘high’ protein expression with concentrations > 75% was demonstrated in cancerous samples, while ‘low’ to ‘medium’ protein expression with 25% - <75 concentrations was shown in normal liver samples. We then validated the PSMA1, PSMA5, and PSMB2 overexpression in the HCCDB database. The results depicted in Figure 2(H–J) confirmed that our target genes were considerably overexpressed in HCC tumor samples compared to noncancerous tissues.

### Association of mRNA expression levels of PSMA1, PSMA5, and PSMB2 with pathological characteristics of HCC patients

The levels of expression of PSMA1, PSMA5, and PSMB2 were evaluated utilizing UALCAN according to the criteria established by the American Joint Committee on Cancer (AJCC) across various pathological stages and grades of HCC. A notable increase in the transcripts of PSMA1, PSMA5, and PSMB2 was observed among HCC patients with differing cancer stages and tumor grades. The expression of these target genes exhibited significant correlations with each cancer stage and pathological tumor grade, except for PSMA5 and PSMB2 when comparing normal versus stage 4. When comparing the target genes within their respective groups, a significant association was observed between cancer stages, pathological grades, and the target genes. These findings are illustrated in Figure 2 alongside their corresponding significance values for clarity. These data suggest that the investigated genes may be potential prognostic indicators for HCC.

### Prognostic values of PSMA1, PSMA5 and PSMB2 in HCC patients

**Figure 3.**
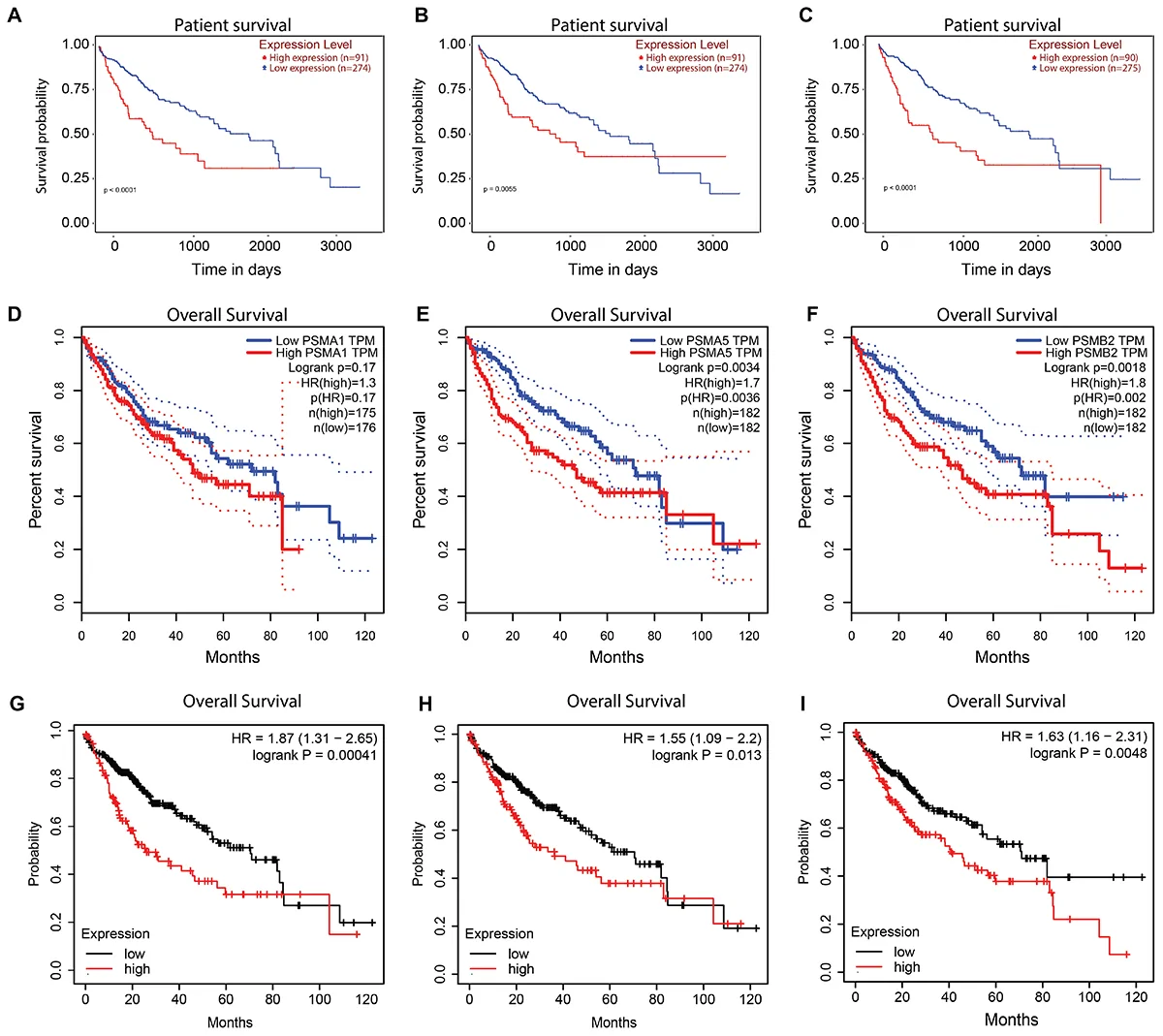
(A-C) The KM-curve for overall survival of the patients, stratified according to the high or low expression levels of PSMA1, PSMA5, and PSMB2 in HCC patients from the UALCAN database. (D-F) the KM-curve for overall survival in the LIHC cohort from the GEPIA database was stratified by high or low PSMA1, PSMA5, and PSMB2 expression levels. (G-I) the Kaplan–Meier curve for overall survival and differential expression of PSMA1, PSMA5, and PSMB2 from TCGA-LIHC cohort.

The prognostic role of the target genes was investigated using LIHC datasets from the UALCAN and GEPIA databases. The high PSMA1, PSMA5, and PSMB2 expression levels in HCC, as shown in Figure 3(A–F), suggested significant negative overall survival (*p<*0.05), respectively. We then validated the prognosis of candidate genes in the KM-Plotter database, and the findings in Figure 3(G–i) revealed that PSMA1, PSMA5, and PSMB2 overexpression was significantly related to shorter overall survival in HCC patients (*p<*0.05). Thus, it was confirmed that there is a negative correlation between PSMA1, PSMA5, and PSMB2 and the overall survival of patients with HCC.

The present study examines the prognostic significance of target genes utilizing LIHC datasets sourced from the UALCAN and GEPIA databases. Elevated levels of PSMA1, PSMA5, and PSMB2 expressions within HCC are depicted in Figure 3(A–F), implying a notable negative impact on overall survival (*p<*0.05). We further corroborated these findings by validating the prognostic implications of the identified candidate genes in the KM-Plotter database. The results depicted in Figures 3(G–I) indicate a significant association between overexpression of PSMA1, PSMA5, and PSMB2 and diminished overall survival among HCC patients (*p<*0.05). Consequently, our study confirms a detrimental correlation between elevated levels of PSMA1, PSMA5, and PSMB2 expression and the overall survival outcomes of individuals afflicted with HCC.

### Co-expression analysis of PSMA1, PSMA5, and PSMB2 in HCC

**Figure 4.**
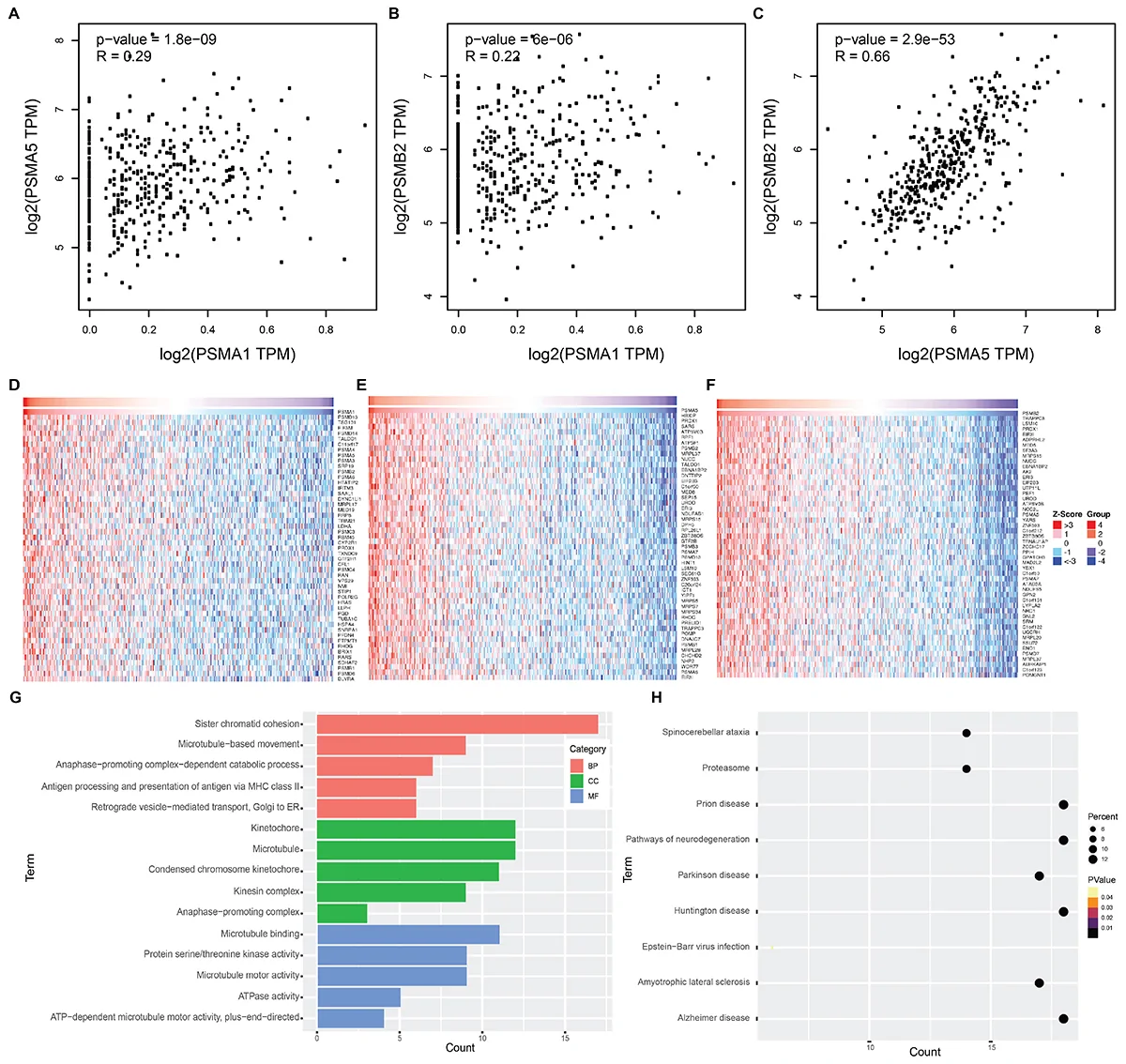
Correlation between the expression levels of (A) PSMA1 and PSMA5, (B) PSMA1 and PSMB2, and (C) PSMA5 and PSMB2 in LIHC from the GEPIA database. (D-F) top 50 positively correlated genes with PSMA1, PSMA5, and PSMB2 in HCC data. (G-H) significantly enriched GO annotations and KEGG pathways in the LIHC cohort for PSMA1, PSMA5, and PSMB2. GO, gene Ontology; BP, biological process; CC, cellular Component; MF, molecular function; KEGG, the Kyoto encyclopedia of genes and genomes.

We next analyzed the correlation between PSMA1, PSMA5, and PSMB2 expression levels in HCC with GEPIA. The results presented in Figure 4(A–C) showed that the expression levels of our target genes are significantly positively correlated with each other: PSMA1 and PSMA5 (*r* = 0.29, *p=*1.8e-9), PSMA1 and PSMB2 (*r* = 0.22, *p=*6e-6), while PSMA5 and PSMB2 (*r* = 0.66, *p=*2.9e-53). The positively correlated genes with PSMA1, PSMA5, and PSMB2 were analyzed in LIHC-cohort with the LinkedOmics database. The heat maps indicate that the top 50 positively correlated genes with PSMA1, PSMA5, and PSMB2 (Figure 4(D–F)). These findings support the existence of a substantial positive correlation between PSMA1, PSMA5, and PSMB2 in HCC.

### Functional annotation and pathway enrichment analysis of co-expressed genes

The top genes list retrieved from LinkedOmics was utilized to assess GO and KEGG analyses. After combining genes displaying co-expression with PSMA1, PSMA5, and PSMB2, 147 co-expressed genes were delineated following the elimination of duplicate entries. The representative GO terms of Biological Processes (BPs), Cellular Components (CCs), and molecular Functions (MFs) were showcased in Figure 4(G). Based on the lowest *p*-value and highest count of genes, sister chromatid cohesion, kinetochore, and microtubule binding were shown to be the most critical BPs, CCs, and MFs, respectively. KEGG analysis showed that our target genes were enriched in pathways in neurodegeneration, prion disease, and proteasome. The top nine KEGG pathways list is depicted in Figure 4(H).

### Promoter methylation of PSMA1, PSMA5, and PSMB2

**Figure 5.**
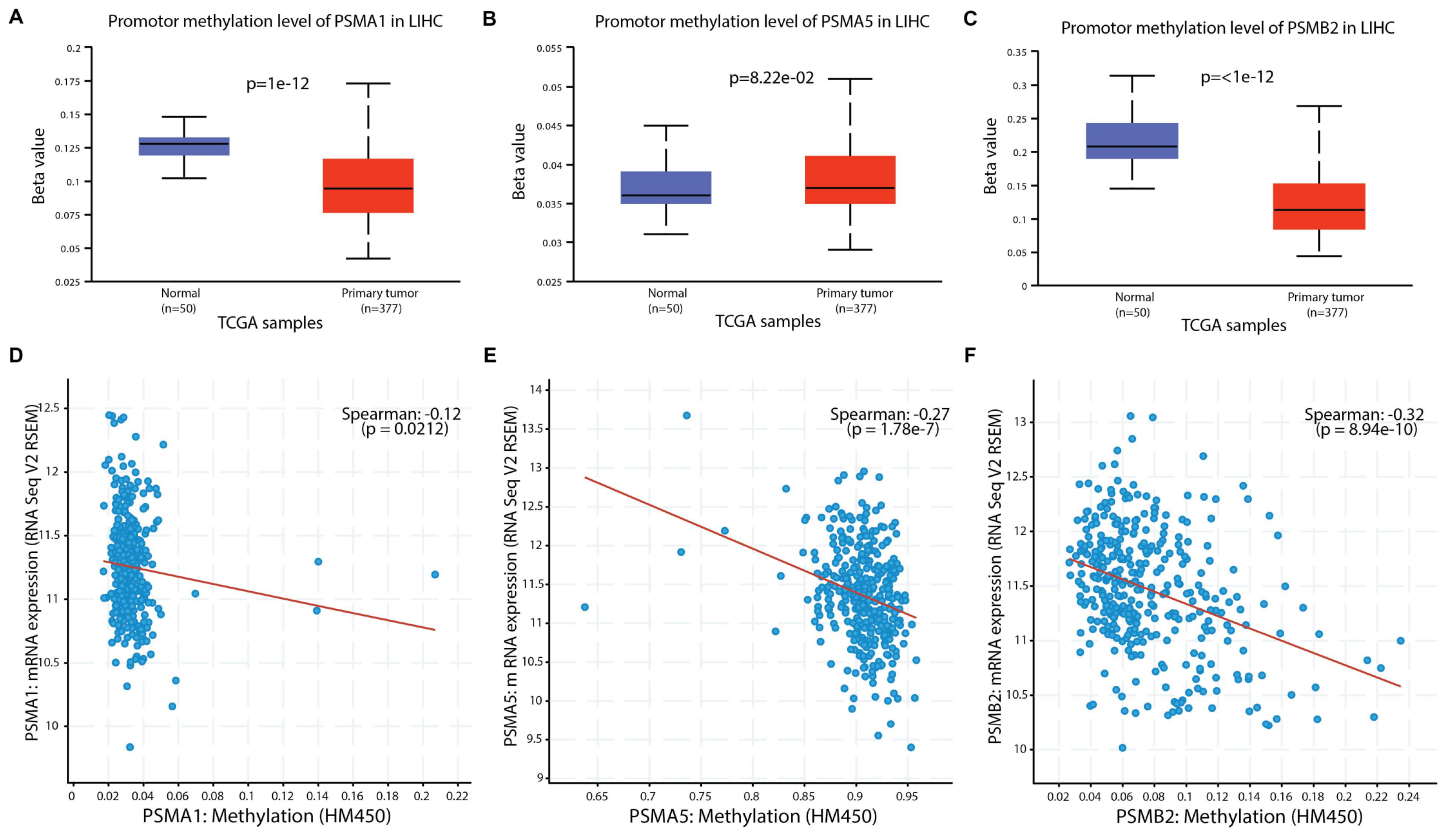
UALCAN analysis of relative promoter methylation of (A) PSMA1, (B) PSMA5, and (C) PSMB2 in normal vis-a-vis HCC. The graphs show the Spearman correlation between (D) PSMA1, (E) PSMA5, and (F) PSMB2 expression levels and DNA methylation in liver HCC from the c-Bioportal.

The LIHC dataset sourced from TCGA was analyzed using the UALCAN database to compare the promoter methylation profiles of PSMA1, PSMA5, and PSMB2 in HCC tumors vis-à-vis normal liver tissue specimens. Our findings demonstrated a significant alteration in the methylation status within the promoter regions of the specified genes. Specifically, PSMA1 and PSMB2 exhibited significant hypomethylation in tumor tissues compared to their counterparts in normal liver tissue specimens (*p* < 0.05). Although a reduction in methylation levels was observed in PSMA5, statistical significance was not attained (*p* = 0.08). These outcomes are visually represented in Figure 5(A–C) to facilitate comprehension. Furthermore, leveraging c-Bioportal, we investigated the interplay between the methylation status of the target genes and their corresponding mRNA expression levels in HCC. Our analysis revealed a notable negative correlation (*p* < 0.01) between the extent of methylation within the target genes and their mRNA expression levels (depicted in Figure 5(D–F)). This observed correlation suggests that hypomethylation within their promoter regions potentially influences the upregulation of PSMA1, PSMA5, and PSMB2 genes.

### Genomic alterations of PSMA1, PSMA5 and PSMB2 in HCC

**Figure 6.**
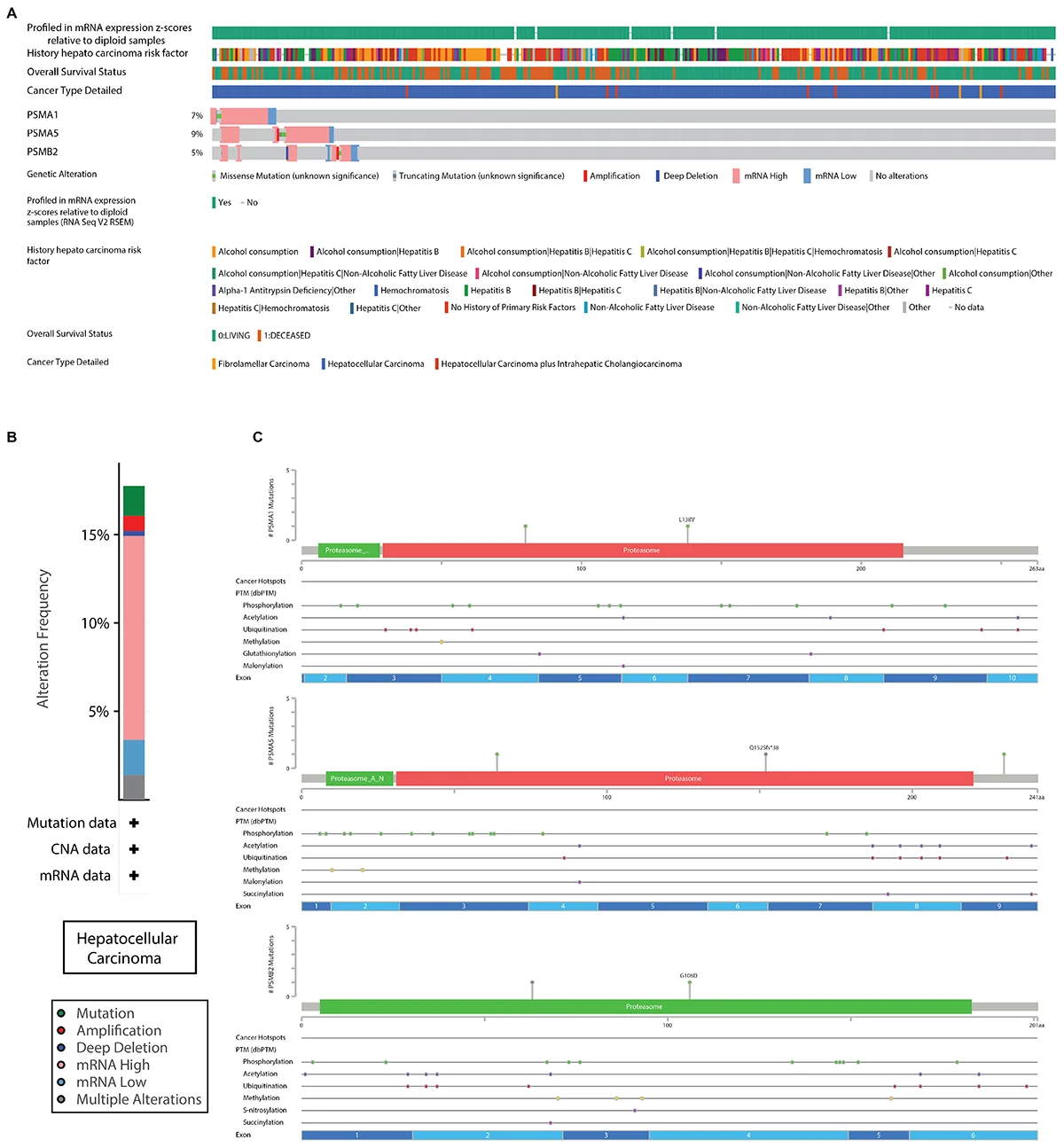
Genomic alterations of PSMA1, PSMA5, and PSMB2 in HCC. (A) an oncoprint of PSMA1, PSMA5, and PSMB2 in HCC, (B) summary of alteration frequency for HCC. (C) mutation type details of PSMA1, PSMA5, and PSMB2 found in HCC.

Utilizing the cBioPortal database, an oncogenic representation was constructed to elucidate the mechanisms underlying the upregulation of PSMA1, PSMA5, and PSMB2 in HCC. Analysis was conducted on a TCGA-LIHC dataset comprising 379 samples to characterize copy number alterations. PSMA1, PSMA5, and PSMB2 exhibited alterations in 7%, 9%, and 5% of individuals with liver HCC, respectively. Notably, HCV emerged as a prominent risk factor associated with the genomic modifications observed in the genes, as discerned from the ‘History of HCC risk factors’ profiling. Moreover, PSMA1, PSMA5, and PSMB2 demonstrated substantial overexpression in HCC compared to HCC with intrahepatic cholangiocarcinoma, correlating with shortened overall survival among patients analyzed for ‘cancer type details’ and ‘overall survival status,’ respectively (Figure 6(A)). Predominantly, mRNA overexpression (11.55%) constituted the most frequent genomic alteration, followed by mutation (1.69%), with amplification (0.85%) occurring less frequently (Figure 6(B)). Mutations identified included missense mutations 180 V and L138V in PSMA1, missense mutations T230S and 164T, and frameshift deletion Q152Sfs *38 in PSMA5, while frameshift deletion N63Tfs *38 and missense mutation G106D were observed in PSMB2 (Figure 6(C)).

### Correlation between copy number alteration and the expression of PSMA1, PSMA5, and PSMB2

**Figure 7.**
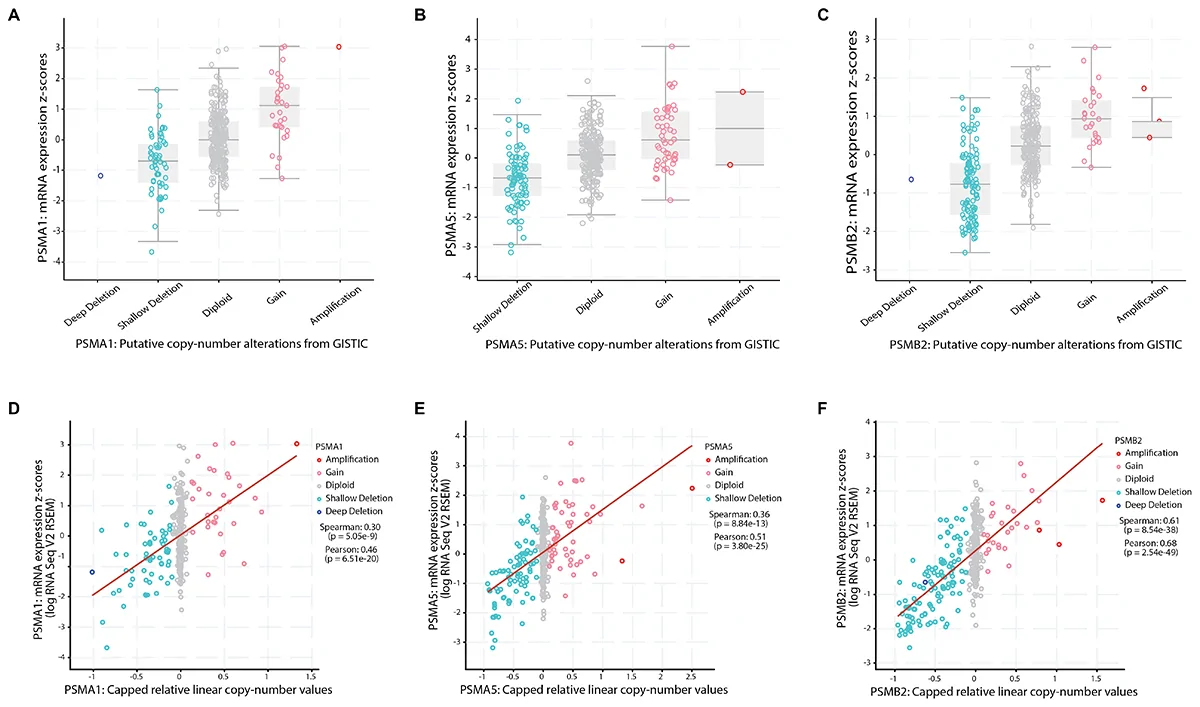
Association between PSMA1 (A), PSMA5 (B), and PSMB2 (C) and discrete copy number alterations. (D-F) Log2 copy numbers of PSMA1, PSMA5, and PSMB2 respectively.

The data from the cBioPortal database demonstrated a significant variance in PSMA1, PSMA5, and PSMB2 copy number alterations in HCC. Our examined genes’ altered copy numbers indicated a rise in level from deep deletion to amplification (Figure 7(A–C)). The expression level of our target genes and the log2 copy number value showed a statistically significant positive connection, with R Spearman values of 0.30, *p=*5.05e-9; R 0.36, *p=*8.84e-13, and R 0.61, and *p=*8.54e-38 for PSMA1, PSMA5, and PSMB2, respectively. The graphs are presented in Figure 7(D–F) for a better understanding.

### PPI network construction of HCV target genes

**Figure 8.**
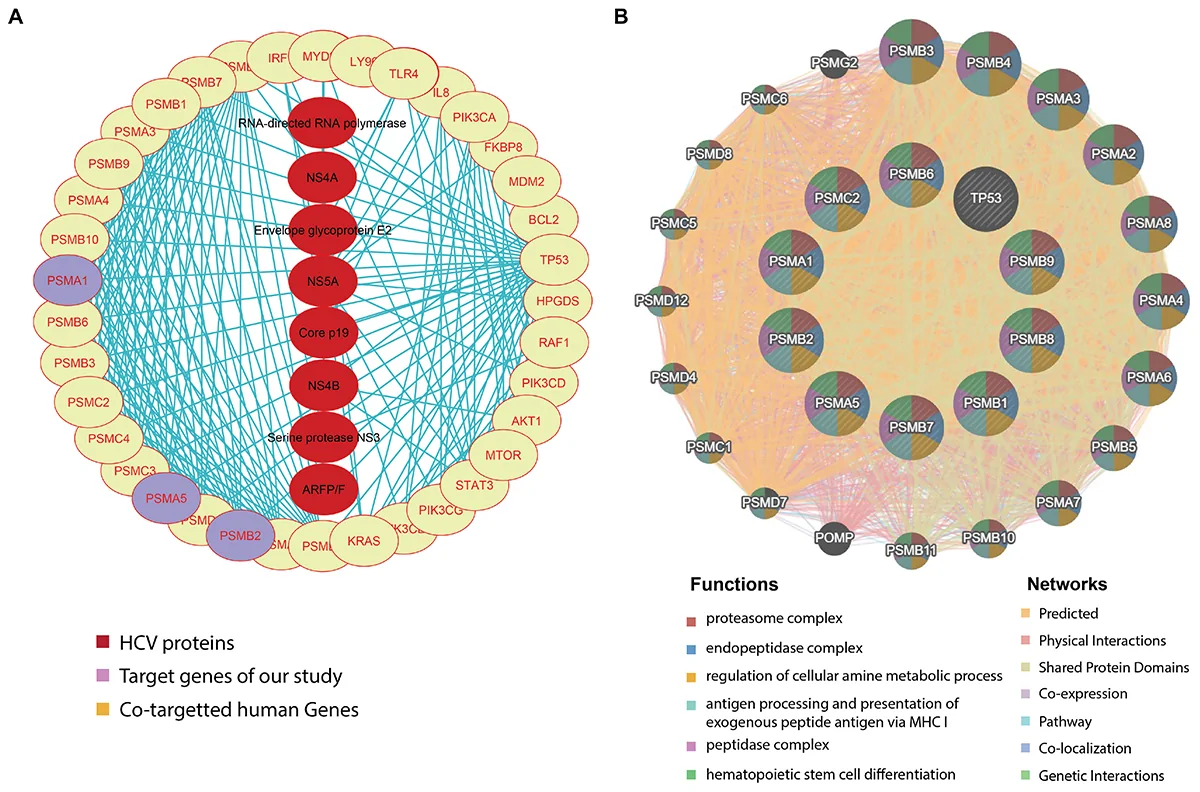
(A) network of virus host protein interactions from STRING viruses database, (B) PPI network of the top 10 target genes of HCV by GeneMANIA database.

We explored the STRING Viruses database to construct a PPI network of HCV proteins with its host-targeted genes. For better understanding, the data were retrieved and visualized with Cytoscape 3.9.1. software. The PPI network of the top 40 target genes of eight HCV proteins (RNA-directed RNA polymerase, NS4A, Envelope glycoprotein E2, NS5A, Core p19, NS4B, Serine protease NS3, and ARFP/F) is depicted in Figure 8(A). The top 10 host genes were selected for further analysis based on the degree of centrality. Using GeneMANIA, a subnetwork of PPI was constituted; the network depicted in Figure 8(B) represents a variety of physical and functional interactions with multiple colors.

### Molecular docking of human receptors and viral proteins

**Figure 9.**
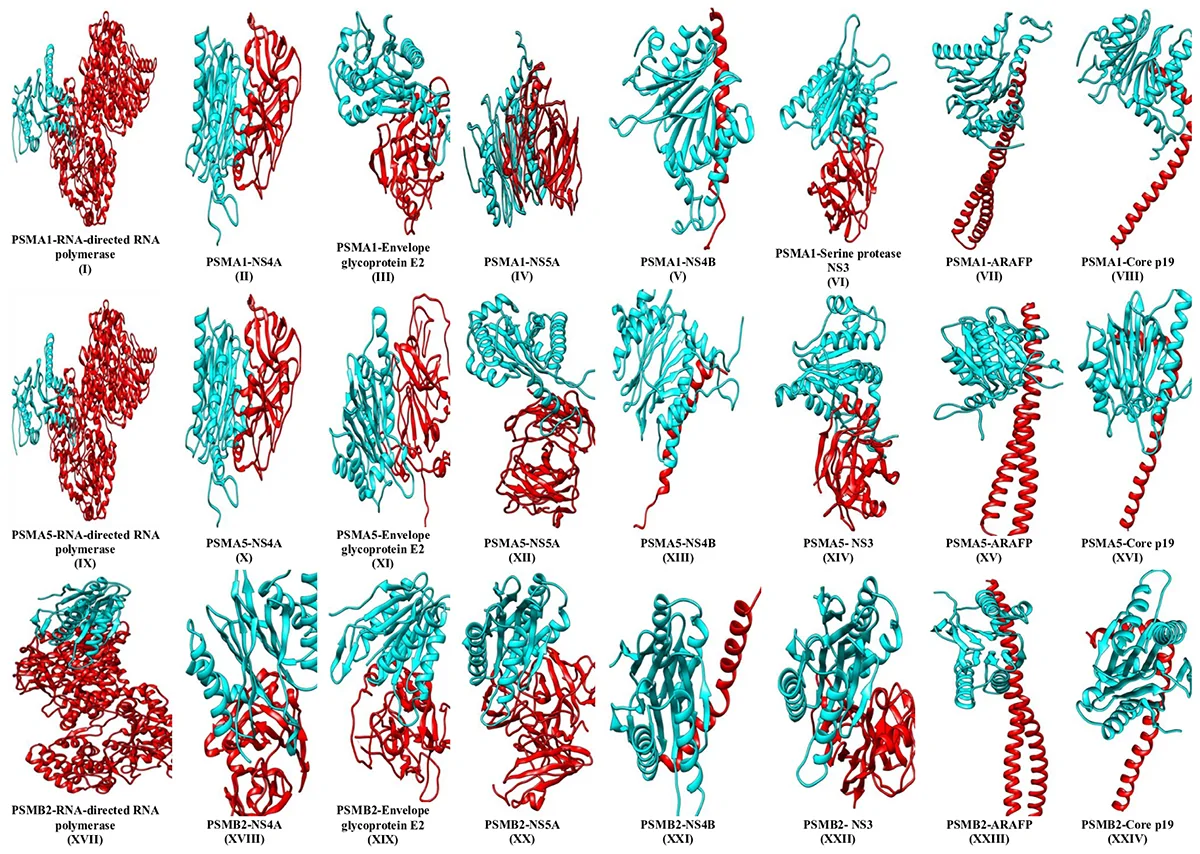
The 3D representation of human receptor proteins (I-VIII) PSMA1, (IX-XVI) PSMA5, and (XVII-XXIV) PSMB2 with viral proteins using the USCF chimera.

The molecular docking was performed to analyze the interaction between human receptors with viral proteins. The objective was to study the coupling junction of viral protein with human receptors, i.e., PSMA1, PSMA5, PSMB2. The intermolecular interactions of each viral protein with human receptors were analyzed using ligplot^+^. The interaction map shows the presence of hydrogen and hydrophobic interactions which depict the strong binding potential between these proteins. The residues involved in hydrogen and hydrophobic interactions are presented in Supplementary Table  S2. It has been observed that Arg157, Asn152, Tyr153, Glu173, Met159, Phe154, and Glu23 are the essential residues of PSMA1 for interaction with most of the viral proteins. Likewise, for PSMA5 Asp127, Gln122, Ser117, Gln114, Asp9, Glu126, and Thr161 are important residues for binding. In PSMB2, residues such as Asp119, Tyr134, Glu49, Arg170, Leu121, Ala131, Ile172, Ala136, and Asn101 play a crucial role in binding to the viral proteins. Table 1 provides a quantitative overview of the interactions present between the complexes. RNA-directed RNA polymerase strongly interacts with PSMA1 by forming four salt bridges, 23 hydrogen bonds, and 253 non-bonding residues. Similarly, NS4A forms five hydrogen bonds, 1 salt bridge, and 101 non-bonding interactions with PSMA1. Similarly, the viral proteins, including Envelope glycoprotein E2, NS5A, NS4B, Serine protease NS3, ARFP1, and Core p19, show strong interaction with PSMA1 by forming 16,18,13,20,4,9 hydrogen bonds, respectively. These docking results are computational predictions of probable virus–host interactions and should be viewed as hypotheses rather than conclusive evidence of physical binding. These anticipated connections will need to be validated experimentally, for example through co-immunoprecipitation or other protein–protein interaction experiments. The diagrammatic illustration of all complexes is represented in Figure 9.

**Table 1.** The interaction energy (ClusPro) of most stable viral proteins and human receptor complexes and interaction analysis using PdbSum.

Complexes	ClusPro	PdbSum		
Human protein-viral protein complex	Interaction energy	Salt bridges	No. of hydrogen bonds	Non-bonding interactions
PSMA1-RNA-directed RNA polymerase	−866.1	4	23	253
PSMA1-NS4A	−850.8	1	5	101
PSMA1-Envelope glycoprotein E2	−954.5	3	16	168
PSMA1-NS5A	−980.9	3	18	189
PSMA1-NS4B	−766.2	2	13	134
PSMA1-Serine protease NS3	−769.6	2	20	175
PSMA1-ARAFP	−849.3	2	4	82
PSMA1-Core p19	−958.3	1	9	138
PSMA5-RNA-directed RNA polymerase	−1029.2	6	18	169
PSMA5-NS4A	−741.6	1	6	97
PSMA5-Envelope glycoprotein E2	−1037.8	2	10	156
PSMA5-NS5A	−915.4	4	5	112
PSMA5-NS4B	−797.9	2	7	121
PSMA5-Serine protease NS3	−881.5	3	15	162
PSMA5-ARAFP	−827.2	1	1	72
PSMA5-Core p19	−1099.7	2	7	140
PSMB2-RNA-directed RNA polymerase	−845.3	2	14	172
PSMB2-NS4A	−756.9	1	5	86
PSMB2-Envelope glycoprotein E2	−1114.2	6	17	225
PSMB2-NS5A	−1019.1	2	11	192
PSMB2-NS4B	−839.0	3	6	144
PSMB2-Serine protease NS3	−905.7	3	16	162
PSMB2-ARAFP	−943.2	2	7	119
PSMB2-Core p19	−1056.7	1	8	126

### Correlation between the expression of HCV target genes and PSMA1, PSMA5, and PSMB2

**Figure 10.**
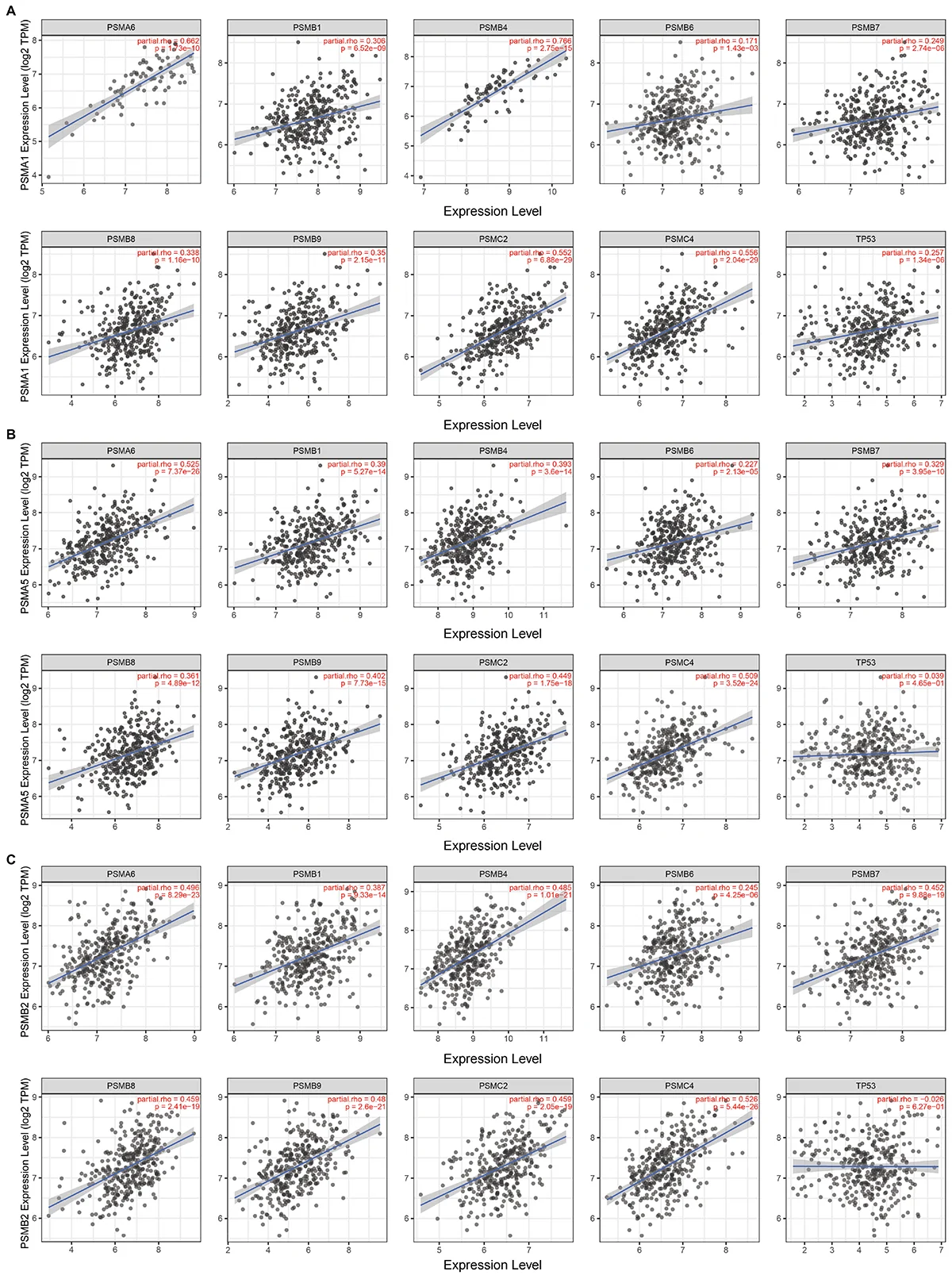
Correlation between the top 10 HCV target genes and (A) PSMA1, (B) PSMA5, and (C) PSMB2 utilizing the TIMER database.

The correlation module within the TIMER database was employed to elucidate the additional correlation between the principal target genes of HCV and PAMA1, PSMA5, and PSMB2. The top decile of genes exhibited a notably positive correlation with PAMA1, PSMA5, and PSMB2, excluding TP53’s correlation with PSMA5 and PSMB2. For enhanced comprehension, scatter plots representing the correlation of each gene are depicted in Figure 10.

### Expression of PSMA1, PSMA5, and PSMB2 following HCV-Core protein expression

**Figure 11.**
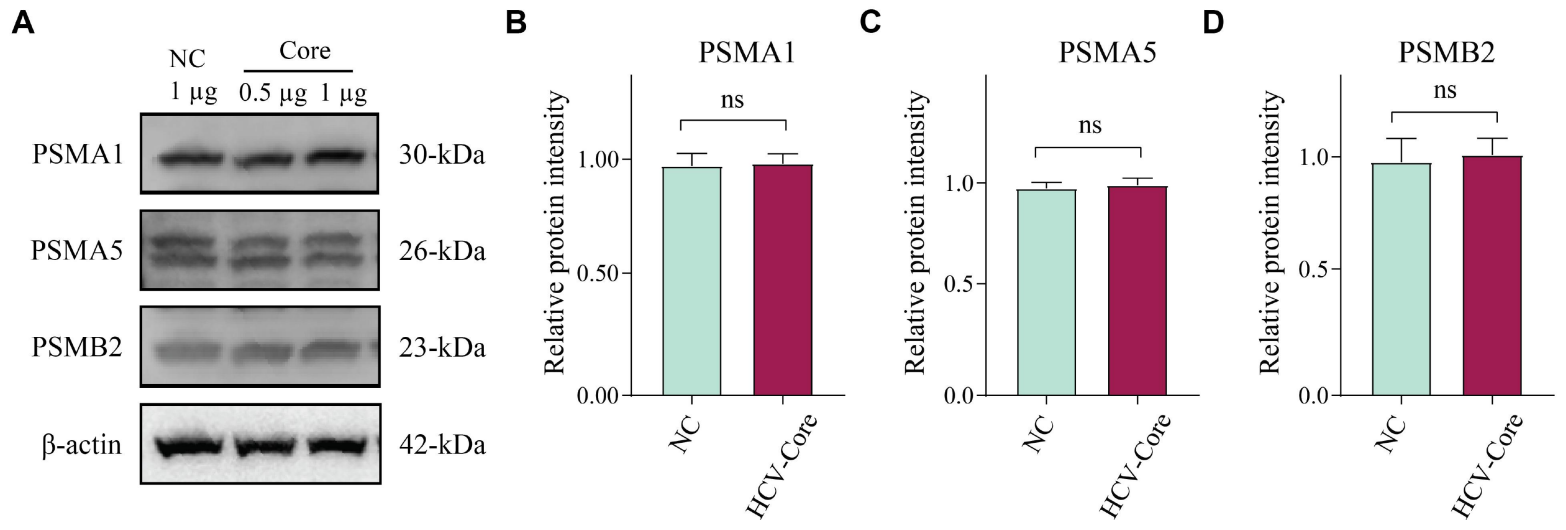
The protein expression changes of PSMA1, PSMA5, and PSMB2 following transfection of HCV-Core in HepG2 cells. (A) WB images showing the protein band intensities of PSMA1, PSMA5, and PSMB2, and (B-D) the corresponding bar graphs represent the quantitative analysis in HepG2 cells. The same cell lysates were used across all blots. The results are expressed as mean ± SD for three independent experiments. Data were analyzed using Student’s t-test. Statistical significance is indicated as ns: not significant.

We investigated if HCV-Core protein expression changes the expression levels of PSMA1, PSMA5, and PSMB2 in HepG2 cells as the molecular docking suggested PPIs between HCV proteins and these proteins. According to WB results depicted in Figure 11, the expression levels of PSMA1, PSMA5, and PSMB2 after HCV-Core protein expression did not differ significantly from the control group.

### Correlation of PAMA1, PSMA5, and PSMB2 with immune infiltration in tumor microenvironment of HCC

**Figure 12.**
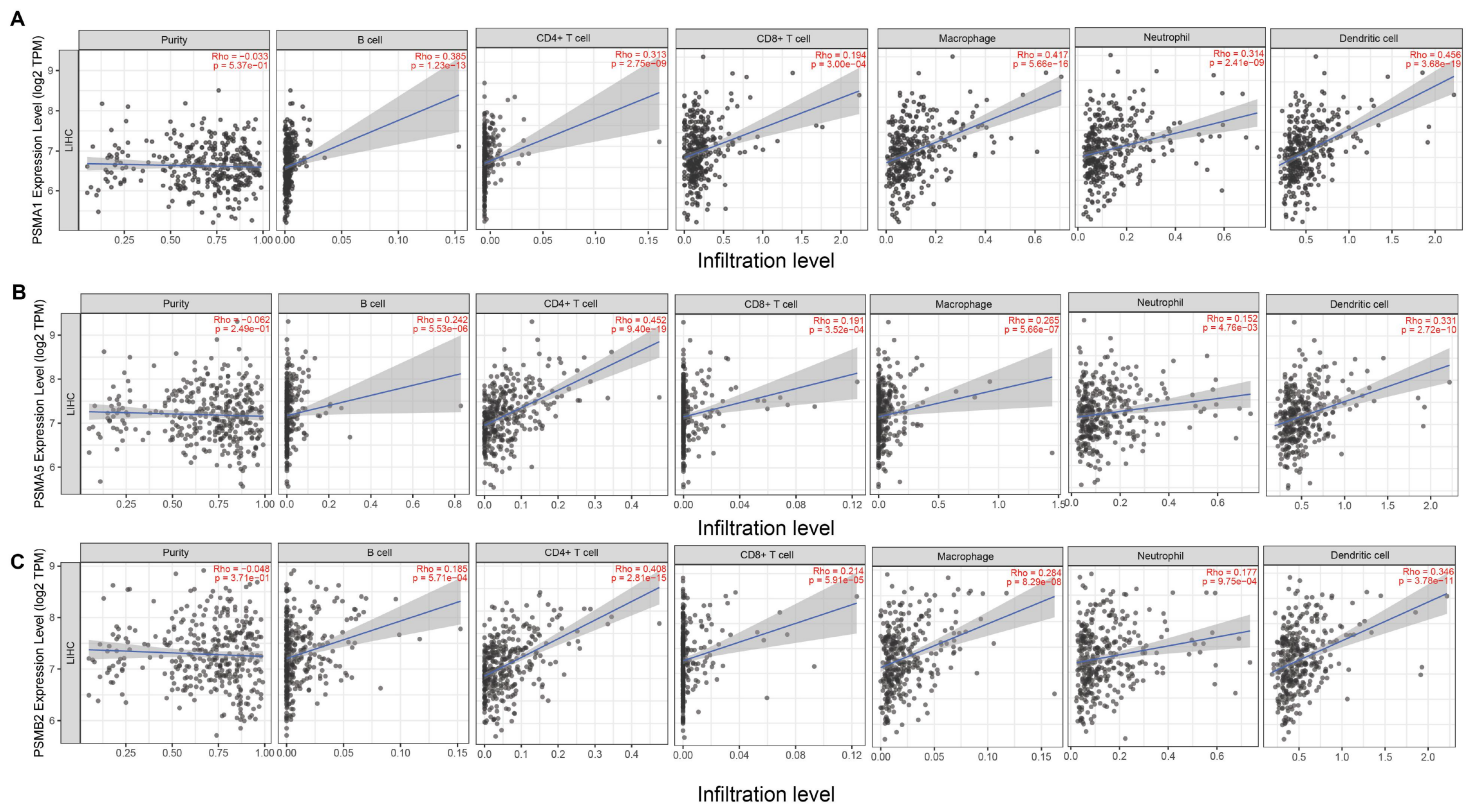
Correlation between (A) PAMA1, (B) PSMA5, and (C) PSMB2 and infiltration of various immune cells in tumor microenvironment from TIMER database.

We employed the TIMER database to assess the association between PSMA1, PSMA5, PSMB2, and immune infiltration. Our analysis revealed a positive correlation between the upregulation of each of these genes and the level of immune cell infiltration within the HCC tumor microenvironment. Notably, PSMA1 expression was positively correlated with infiltration levels of B cells (*r* = −0.385, *p=*1.23e-13), CD4+T cells (*r* = 0.313, *p=*2.75e-09), CD8+T cells (*r* = 0.194, *p=*3.00e-04), macrophages (*r* = 0.417, *p=*5.66e-16), neutrophils (*r* = 0.314, *p=*2.41e-09), and dendritic cells (*r* = 0.456, *p=*3.68e-19). The PSMA5 expression was positively correlated with the infiltration levels of B cells (*r* = 0.242, *p=*5.53e-06), CD4+T cells (*r* = 0.452, *p=*9.40e-19), CD8+T cells (*r* = 0.191, *p=*3.52e-04), macrophages (*r* = 0.265, *p=*5.66e-07), neutrophils (*r* = 0.152, *p=*4.76e-03), and dendritic cells (*r* = 0.331, *p=*2.72e-10). The expression of PSMB2 was positively correlated with infiltration levels of B cells (*r* = 0.185, *p=*5.71e-04), CD4+T cells (*r* = 0.408, *p=*2.81e-15), CD8+T cells (*r* = 0.214, *p=*5.91e-05), macrophages (*r* = 0.284, *p=*8.29e-08), neutrophils (*r* = 0.177, *p=*9.75e-04), and dendritic cells (*r* = 0.346, *p=*3.78e-11). The scatter plots for each gene are depicted in Figure 12. These findings indicate that our target genes might play crucial roles in infiltrating various immune cells in the tumor microenvironment of HCC.

### Correlation between the expression levels of PSMA1, PSMA5, and PSMB2 and different immune markers

Utilizing the TIMER database’s correlation module, we conducted a comprehensive examination into the interrelationship between the expression levels of PSMA1, PSMA5, and PSMB2 and immune markers pertinent to distinct subsets of immune cells in Hepatocellular Carcinoma (HCC). The findings delineated in Table 2 elucidate a statistically significant correlation between the expression levels of most immune cell gene markers and the genes under investigation. Particularly noteworthy is the robust correlation between the gene markers associated with Monocytes, M2 macrophages, and tumor-associated macrophages (TAMs) with PSMA1, PSMA5, and PSMB2 expression levels in LIHC, except for M1 macrophages with PSMB2. These findings strongly imply the involvement of PSMA1, PSMA5, and PSMB2 in the process of macrophage polarization within the context of HCC. Furthermore, the expression levels of the majority of gene markers of B cells, Dendritic cells (DCs), Neutrophils, PDL1, T-cell exhaustion, T helper 1 (Th1), T helper 2 (Th2), and Treg cells exhibited a significant correlation with the expression levels of the target genes in HCC, except for natural killer cells (NKs). These compelling observations substantiate the assertion that PSMA1, PSMA5, and PSMB2 are intricately linked with immune infiltration in HCC, thus implying their potential involvement in immune evasion mechanisms.

**Table 2.** PSMA1, PSMA5, and PSMB2 correlation with gene markers of innate and adaptive immune cells in LIHC.

Description	Gene Marker	PSMA1		PSMA5		PSMB2	
		Correlation	*p*-Value	Correlation	*p*-Value	Correlation	*p*-Value
Monocyte	CD86	0.412	***	0.32	***	0.302	***
	CD115	0.352	***	0.319	***	0.28	***
M1 Macrophage	NOS2	0.122	*	−0.072	0.183	0.025	0.644
	ROS1	0.178	***	0.007	0.901	0.074	0.17
M2 Macrophage	CD163	0.289	***	0.239	***	0.195	***
	VSIG4	0.315	***	0.327	***	0.279	***
	CSF1R	0.352	***	0.319	***	0.28	***
TAM	CCL2	0.24	***	0.034	0.5	0.117	*
	CD68	0.306	***	0.238	***	0.224	***
	IL10	0.302	***	0.381	***	0.186	***
DC’s	CD11C (ITGAX)	0.408	***	0.254	***	0.205	***
	CD1C	0.183	***	0.009	0.871	−0.019	0.725
	NRP1	0.396	***	0.114	*	0.174	**
Neutrophils	CCR7	0.16	***	−0.05	0.351	0.01	0.851
	ITGAM	0.412	***	0.369	***	0.378	***
	CD59	0.391	***	0.172	**	0.187	***
Th1	STAT1	0.495	***	0.269	***	0.245	***
	STAT4	0.271	***	0.123	*	0.07	0.194
	TBX21	0.108	*	0.032	0.557	0.026	0.635
	CD4	0.279	***	0.192	***	0.095	0.076
	IFNG	0.203	***	0.205	***	0.189	***
Th2	GATA3	0.27	***	0.106	*	0.138	*
	STAT6	0.317	***	0.169	**	0.154	**
	CXCR4	0.419	***	0.132	*	0.192	***
	CCR4	0.357	***	0.065	0.226	0.05	0.354
Treg	FOXP3	0.245	***	0.084	0.121	0.124	*
	CCR8	0.427	***	0.206	***	0.181	***
	STAT5B	0.394	***	0.081	0.131	0.079	0.143
	TGFB1	0.334	***	0.084	0.121	0.154	**
NKs	KIR3DL1	0.064	0.238	−0.016	0.771	−0.0004	0.934
	KIR2DL1	−0.014	0.7	−0.035	0.521	−0.032	0.559
	KIR2DS4	0.101	0.061	0.056	0.3	0.02	0.706
B Cell	CD19	0.201	***	0.126	*	0.148	**
	CD79A	0.082	0.128	−0.037	0.491	−0.049	0.366
	KRT20	0.247	***	0.306	***	0.23	***
T-cell exhaustion	PD1 (PDCD1)	0.242	***	0.082	0.127	0.146	**
	CTLA4	0.287	***	0.206	***	0.182	***
	TIM3	0.413	***	0.342	***	0.307	***
	LAG3	0.166	**	0.091	0.092	0.103	0.055
	GZMB	0.046	0.397	0.05	0.354	0.051	0.341
PDL1	CD274	0.414	***	0.163	**	0.269	***

## Discussion

Proteasomes are ubiquitously present in eukaryotic cells and are the principal enzymatic apparatus responsible for intracellular protein degradation [8]. This proteolytic system exerts regulatory control over numerous vital proteins implicated in the pathogenesis of various malignancies and immunological disorders [18,19]. A mounting body of evidence underscores the association between proteasome subunit (PSM) genes and cancer, elucidating their involvement in pivotal cellular processes such as apoptosis, cell cycle regulation, DNA repair, invasion, metastasis, and proliferation in cancer cells [11]. Proteasome inhibitors, such as bortezomib, carfilzomib, and ixazomib, have demonstrated significant efficacy in treating multiple myeloma. The proteasome inhibitor is a type of medication that inhibits the activity of proteasomes, leading to the accumulation of damaged or unwanted proteins within cells. This accumulation can disrupt various cellular processes, ultimately leading to cell death. Elevated expression levels of various PSM genes have been consistently observed in tumor tissues compared to adjacent normal tissues in various malignancies, including HCC. For instance, increased 26S proteasome activity has been documented in prostate cancer [10], while Li et al. reported upregulation of PSMA1-7 expression in breast, bladder, lung, gastric, head, and neck cancers compared to normal tissues [11,12]. Consequently, further investigation into proteasome-related genes’ carcinogenic and immune regulatory roles became imperative. In the present study, we aimed to elucidate the involvement of PSMs in developing prognostic biomarkers for HCC. We identified PSMA1, PSMA5, and PSMB2 as potential biomarkers for HCC through comprehensive omics analyses.

Notably, the identification of PSMA1, PSMA5, and PSMB2 suggests that not all proteasome subunits contribute equally to HCC progression. PSMA1 and PSMA5 are structural α-ring subunits that regulate proteasome assembly and substrate gating, thereby controlling substrate access to the catalytic chamber and maintaining proteasome stability. In contrast, PSMB2 is a structural β-ring subunit that contributes to the integrity and proper organization of the 20S catalytic core. Unlike catalytic β subunits such as PSMB5, which directly mediate proteolysis and are established therapeutic targets of proteasome inhibitors, PSMA1, PSMA5, and PSMB2 primarily maintain proteasome architecture and function. Their dysregulation may therefore enhance global proteasome efficiency, accelerating the degradation of tumor suppressors and cell-cycle regulators that favor malignant transformation.

In the current study, we tended to assess the expression levels of PSMA1, PSMA5, and PSMB2 in HCC. Our findings indicate a notable upregulation of all three genes in HCC tumor specimens compared to their corresponding adjacent normal liver tissues. These outcomes find support from many previously conducted studies documented in scholarly literature. For instance, PSMA1 has been documented to exhibit distinct expression patterns in colon cancer and liver metastasis and serves as a cancer-specific immunogen, functioning as a marker for cancer [20]. Reports have also highlighted the overexpression of PSMA1 in pulmonary neuroendocrine tumors [8], colorectal cancer tissue [21], and gastric cancer [22,23], relative to normal tissue samples. While PSMA1 has been implicated as an oncogene in various human cancers [23], its precise role as a pivotal oncogene in HCC remained unelucidated. In our current investigation, we have revealed its overexpression and genomic alterations within HCC tumors. Beyond its altered expression, PSMA1 plays an essential role in α-ring assembly and regulates substrate entry into the proteasome core. Consequently, aberrant PSMA1 expression may enhance proteasome-mediated degradation of proteins involved in cell-cycle arrest and apoptosis, thereby promoting tumor progression. Likewise, existing literature corroborates our findings regarding PSMA5, which has been observed to be overexpressed in pulmonary neuroendocrine tumors [8], prostate cancer [10], as well as breast and bladder cancers [12], compared with corresponding normal tissues. PSMA5 likewise contributes to α-ring stability and efficient assembly of the 20S proteasome. Its overexpression may increase overall proteasome activity, facilitating rapid protein turnover required for sustained proliferation of cancer cells. Moreover, reports have documented the overexpression of PSMB2 in clear-cell renal cell carcinoma [14], gastric cancer [19], osteosarcoma [24], and HCC [25]. Unlike catalytic β subunits responsible for proteolytic cleavage, PSMB2 primarily supports the structural integrity of the β-ring. Dysregulation of PSMB2 may therefore indirectly increase proteasome efficiency and alter degradation of proteins by controlling proliferation, apoptosis, and stress responses.

Moreover, our study has demonstrated a correlation between the upregulation of these genes and diminished overall survival rates among HCC patients. The prognosis of patients with HCC depends on several variables, including the extent to which neoplastic growth has spread throughout the body and the histological differentiation of their tumors. Thus, we correlated the PSMA1, PSMA5, and PSMB2 expression levels with distinct pathological tumor grades and cancer stages in HCC to evaluate their clinical importance. Our findings revealed an elevation in the expression levels of PSMA1, PSMA5, and PSMB2 across varying tumor grades and cancer stages, underscoring the pivotal role of these genes in the initiation and advancement of tumorigenesis in HCC. It is imperative to note that the prognosis of HCC is closely related to its staging. Nevertheless, disease-free survival and overall survival in HCC patients at the same stage have been reported to be different [26]. This evidence supports the role of PSMA1, PSMA5, and PSMB2 in the prognosis of HCC patients. PSMA1, PSMA5, and PSMB2 exhibit potential as formidable prognostic biomarkers, presenting avenues for tailored therapeutic interventions in HCC. Their strong association with distinct cancer stages underscores their promise as targets for personalized therapeutic approaches.

Genomic alteration and mutation are the major inducers for the initiation and development of several cancers [16]. Demethylation of signaling-related and carcinogenic genes is a major source of hepatic carcinogenesis caused by HCV infection. Emerging evidence suggests that HCV can modulate gene expression via epigenetic mechanisms by impacting various host factors, with a substantial proportion of liver cancer (90%) cases potentially evolving through the perturbation of intricate epigenetic networks [27]. Oncogenic viral infections typically disrupt DNA repair mechanisms and perturb genetic and epigenetic equilibrium [28]. HCV, recognized as an oncogenic virus, fosters carcinogenesis by subjecting the liver to persistent damage and regeneration through direct and indirect molecular mechanisms, including inflammation, proliferation, apoptosis, and genomic alterations [4]. While HCV is considered to contribute to carcinogenesis indirectly, a direct association between chronic HCV infection and HCC development exists, suggesting the virus’s direct role in tumorigenesis via its viral proteins and the creation of a tumorigenic microenvironment within infected cells [29]. Structural and nonstructural proteins of HCV have been documented to dysregulate potentially oncogenic signaling pathways [30]. Virus-induced malignancies are linked to changes in gene methylation. HCV infection significantly contributes to the inactivation of tumor suppressor genes through hypermethylation and the activation of oncogenes via demethylation processes, thereby facilitating carcinogenesis [2]. In our current analysis, the overexpression of our target genes is due to a combination of factors, including genomic alterations and DNA hypomethylation. The present investigation anticipates that the regulation of PSMA1, PSMA5, and PSMB2 genes may be influenced by DNA hypomethylation. The findings of this study are corroborated by the extant literature pertaining to methylation and genomic modifications, which indicates that hypomethylation and genomic alterations contribute to the upregulation of numerous genes, including oncogenes [27]. DNA methylation-related changes, such as hypermethylation, hypomethylation, and imprinting loss, have been linked to various human disorders, including cancer [31]. Although promoter hypomethylation of PSMA1 and PSMB2 was associated with their increased expression, the present study does not establish that HCV directly induces hypomethylation of these specific genes. Previous studies have shown that HCV proteins, particularly Core and NS5A, can modulate the host epigenetic machinery by altering the expression and activity of DNA methyltransferases (DNMTs), resulting in widespread DNA methylation abnormalities that contribute to hepatocarcinogenesis [27]. Such epigenetic remodeling may provide a plausible mechanism for the aberrant expression of proteasome subunits observed in HCC. However, whether HCV-mediated DNMT dysregulation directly targets the promoters of PSMA1, PSMA5, or PSMB2 remains unknown. Therefore, the association observed in the present study should be considered correlative rather than causal, and future studies investigating DNMT activity and promoter methylation following HCV infection or expression of HCV proteins are warranted.

Utilizing a PPI network analysis, our study identified the genes as direct targets of HCV. Molecular docking analysis further predicted potential binding interactions between viral proteins and human receptors, implying that PSMA1, PSMA5, and PSMB2 possess notable receptivity to viral proteins. Although molecular docking predicted strong interactions between HCV proteins and PSMA1, PSMA5, and PSMB2, HCV-Core expression did not significantly alter their protein levels in HepG2 cells. This finding suggests that the predicted interactions may regulate protein function, localization, stability, or post-translational modifications rather than protein abundance. Alternatively, regulation of these proteasome subunits may require other HCV proteins, different viral genotypes, prolonged infection, or the coordinated action of multiple viral components. Therefore, further studies using additional HCV proteins, infectious HCV models, and HBV-associated HCC models are warranted. Although the HCV-Core transfection experiment provides additional in vitro evidence, it should not be considered independent clinical confirmation. A significant limitation of the current study is the absence of independent clinical validation in a well-characterized HCC population. Further confirmation using independent HCC patient cohorts and various experimental models is required to demonstrate the HCV-specific relevance and clinical applicability of our findings.

In this study, we delved deeper into the immunoregulatory functions of PSMA1, PSMA5, and PSMB2, within the context of HCC. We discovered a substantial association between PSMA1, PSMA5, and PSMB2 expression levels with the infiltration of numerous immune cells and immune gene markers. The association shows that these genes control tumor immunity in HCC [32]. Furthermore, multiple studies have found a link between tumor-infiltrating immune cells and the development of HCC [33]. The immune markers for M2 macrophages, such as CD163, CSF1R, and VSIG4, were positively linked with the expression of our target genes; however, the gene markers for M1 macrophages, such as NOS2 and ROS1, were only correlated with PSM1. Our findings demonstrate the role of PSMA1, PSMA5, and PSMB2 in HCC-associated macrophage polarization. Macrophages, as significant components of the immune infiltrate, have been widely investigated and reported to play critical roles in tumor genesis and progression. M1 macrophages, typically activated, play a role in antitumor immunity. On the other hand, M2 macrophages, which are alternatively activated, display pro-tumorigenic effects and have been linked to a high prevalence of intra-hepatic metastases and poor survival [26]. In virus-associated HCC, there is increased M2-type macrophage polarization in the tumor immunological microenvironment (Y. Z [2].). The HCV core protein influences the development of immune-related cells through a TLR2/STATs signaling pathway and inhibits monocyte development into both M1 and M2 macrophages [34]. In contrast to M1 macrophages, which produce pro-inflammatory cytokines, nitric oxide (NO), or ROS, phagocytose microbial pathogens, and launch an immune response to combat viral infections, M2 macrophages are involved in tissue repair and wound healing when activated by molecules like IL-10 or C1q [35]. The UPS regulates immune homeostasis by modulating antigen processing, inflammatory signaling, and the production of immune checkpoint-associated proteins. As a result, deregulation of structural proteasome subunits such as PSMA1, PSMA5, and PSMB2 may disrupt proteasome activity and immunological signaling, contributing to an immunosuppressive tumor microenvironment that promotes M2 macrophage polarization and T-cell exhaustion. Although the current study only found correlated relationships, the data suggest a reasonable mechanistic theory relating proteasome dysfunction to immune evasion in HCC, warranting further experimental exploration. Furthermore, our investigation reveals that PSMA1, PSMA5, and PSMB2 contribute to the induction of T-cell exhaustion through the activation of Tregs via programmed cell death protein 1 (PD-1), programmed death-ligand 1 PD-L1, TIM3, and CTLA4. The accumulation of Tregs alongside impaired T-cell functionality could impede immune surveillance, thereby fostering tumor initiation and recurrence [36]. These findings suggest that PSMA1, PSMA5, and PSMB2 facilitate immune evasion by recruiting and regulating immune infiltrates in the tumor microenvironment.

While the mechanisms and functions of individual molecular constituents remain incompletely elucidated, immune responses play a pivotal role in progressing chronic hepatitis C virus (CHCV) infection to HCC. Several studies have demonstrated that HCV-driven abnormal expression of the immune system launches the inflammatory and fibrotic process that ultimately results in HCC. Virus-induced immune counterattacks on infected liver cells are primarily responsible for HCV carcinogenesis. Broadly focused CD4+ and CD8+ T cell responses have been linked to eradicating HCV infection [35]. Unfortunately, chronic hepatitis results from the infection when the host cannot get rid of the HCV virion, destabilizing the liver’s immune microenvironment, resulting in immune evasion and contributing to the development of HCC [37]. HCV viruses have numerous proteins; in addition to the involvement in the HCV life cycle, some have exceptional ability to evade the immune response and develop chronicity. HCV core protein has been reported to affect host immune responses by altering various host pathways [35]. Our study suggests that the combination of PSMA1, PSMA5, and PSMB2 expression levels and immune infiltrates might give better prognostic performance, allowing the prediction of the incidence of HCC with high accuracy. Collectively, our findings suggest that PSMA1, PSMA5, and PSMB2 are not merely overexpressed proteasome constituents but represent biologically distinct structural subunits whose dysregulation may alter proteasome assembly, substrate gating, and protein homeostasis. These characteristics distinguish them from other proteasome components and provide a mechanistic basis for their prognostic value in HCV-associated HCC.

## Conclusion

This article reports several new findings. Our investigation demonstrates a notable upregulation of PSMA1, PSMA5, and PSMB2 in HCC, suggesting a potential mechanism by which these proteins attain immunogenicity. Furthermore, we elucidate DNA methylation and genomic alterations as pivotal factors contributing to the aberrant overexpression of these genes. Particularly noteworthy is our discovery of these genes’ prognostic and predictive significance in HCC. Moreover, we observe a correlation between the expression levels of these genes and the various grades and stages of HCC. Additionally, our analysis reveals a positive correlation between immune cell infiltration levels and the expression of PSMA1, PSMA5, and PSMB2, suggesting their involvement in HCC development and progression and the immune modulation of the tumor microenvironment.

Collectively, these findings suggest that dysregulation of specific structural proteasome subunits, rather than proteasome dysfunction in general, may contribute to hepatocarcinogenesis. PSMA1, PSMA5, and PSMB2 therefore represent promising prognostic biomarkers and potential therapeutic targets for HCC, although further experimental studies are required to validate their mechanistic roles and clinical applicability.

### Future perspectives

Future studies should validate the expression and prognostic significance of PSMA1, PSMA5, and PSMB2 in independent HCC patient cohorts using techniques such as RT-qPCR, immunohistochemistry, or WB. In addition, functional in vitro and in vivo studies are required to find the molecular mechanisms by which these structural proteasome subunits contribute to HCC progression and their potential as therapeutic targets.

## Supplementary Material

Supplementary Materials.docx

## Data Availability

All the data is available online.
