# Supplementary material for: PSMA1, PSMA5, and PSMB2 serve as prognostic biomarkers and are correlated with tumor-infiltrating leukocytes in HCC
Source: Epigenetics. 2026 Sep 14;21(1):2726624. doi: 10.1080/15592294.2026.2726624 (PMC13577265; doi:10.1080/15592294.2026.2726624)
Supplement: Supplementary file 1 [file KEPI_A_2726624_SM7095.docx]

**Supplementary Materials**

**Table S1: the RCSB PDB ID of human receptor proteins and viral proteins.**

| Human receptor proteins (PDB-id) chain | Viral protein (PDB-id) chain |
| --- | --- |
| PSMA1 (4R3O) | RNA-directed RNA polymerase (1SCJ) |
| PSMA5 (4R3O) | NS4A (1JXP) |
| PSMB2 (4R3O) | Envelope glycoprotein E2 (6MEJ) |
|  | NS5A (3FQQ) |
|  | NS4B (2LVG) |
|  | Serine Protease NS3 (IJXP) |
|  | ARAFP |
|  | Core p19 |

**Table S2: The molecular interaction between human receptors and viral proteins.**

|  |  | **Human receptor** | **Viral protein** | **Human receptor** | **Viral protein** |
| --- | --- | --- | --- | --- | --- |
| **Human protein-viral protein complex** | **Interaction energy** | **hydrogen bonds** | **hydrogen bonds** | **Hydrophobic interaction** | **Hydrophobic interaction** |
| PSMA1-RNA-directed RNA polymerase | -866.1 | Arg157, Asp155, Tyr153, Asn152, Asn152, Ser150, Gln121, Arg107, Arg107, Ile19, Arg18, Arg18, Trp13, Asp155, Tyr153, Tyr123, Ser110, Ser106, Gln16, Thr11, Asp9, Tyr6 | Ala 5, Gln438, Lys 98, Glu437, Gln436, Asn142, Lys106, Lys98, Arg168, Asn28, Asn24, Gln436, Arg401, Glu398, Arg401, Asp444, Ala435, His402, Ala16, Glu 17, His95 | Glu23, Ser114, Tyr24, His20, Asp7, Gln5, Met159, Val12, Phe154, Pro15, Glu27 | Cys14, Lys441, Ala15, Pro404, Thr403, Val405, Mse139, Lys141, Ile160, Thr399, Ala400, Ser96, Lys100, Ile23, Phe162, Ala140, Val405, Thr403, Pro404, Ala15, |
| PSMA1-NS4A | -850.8 | Arg157, Tyr153, Asn152, Gln16 | Arg24, His110, Ala39, Thr38 | Met140, His143, Lys30, Ser150, Met26, Cys148, Ile161, Gln146, Arg169, Arg18, Ile19, Gly17, Glu23, Glu173 | Gln9, Ile18, Leu21, Thr22, Thr40, Leu13, Cys16, Ser37, Ile17, Leu14, Ser133, Val132 |
| PSMA1-Envelope glycoprotein E2 | -954.5 | Arg157, Asn152, Arg122, Arg107, Arg101, Glu173, Asp155, Gln121, Arg107, Pro15 | Arg630, Arg639, Asn540, Trp420, His474, Glu637, Gly547, Pro545, Arg521, Ala522, Asn417, Trp420, His421 | Ala151, Met26, Lie22, Glu23, Met176, Phe154, Gln146, Cys156, Met159, Arg169, Ile19, Trp13 | Thr542, Asn 541, Gly635, Val633, Val636, His638, Tyr632, Leu546, Pro544 |
| PSMA1-NS5A | -980.9 | Cys156, Gln121, Gln146, Cys148, Glu23, Arg122, Asn152, Asp155, Ser110, Tyr153, Arg157, Glu173, Thr170 | Trp84, Arg78, Asp154, Arg41, Lys44, Phe37, Phe36, Gly33, Cys165, Pro167, His85, Glu171 | Met176, Ala158, Arg169, Glu27, Lys30, Phe145, Phe154, Val129, Ile161, Met26, Met159, Gln166, Gln117, Arg107 | Ser107, Lys166, Trp84, Arg78, Asp154, Arg41, Lys44, Phe36, Phe37, Gly33, Cys165, Pro167, His85, Glu171 |
| PSMA1-NS4B | -766.2 | Glu178, Asn152, Glu181, Asp155, Gly17, Trp13, Thr11, Gln121, Gln117, Arg157 | Gly10, Gln31, Asp34, Ala38, Gln40, Gln33, Lys20, Ser2, Leu24, Arg3 | Thr120, Ile22, Ser150, Tyr153, Phe154, Met180, Ser177, Ile19, Arg18, Tyr6, Val12, Gln5, Tyr123 | Ile13, Gln36, Pro37, Met39, Ala14, Leu17, Leu6, Ala1, Ile21, Ala28, Leu25, Ala32 |
| PSMA1-Serine protease NS3 | -769.6 | Asp139, Arg107, Asn152, Glu173, Cys156, Ser114, Ser110, Asp155, Arg157, Tyr153 | Arg109, Gln8, Ser7, Lys136, Gln9, Gln28, His110, Ser37, Gln41, Thr38, Ala39, Pro131, Ser133, Arg130 | Met140, Ser106, Thr120, Ala151, Ser150, Phe154, His143, Phe145, Glu23, Met26, Val29, Cys148, Gln146, Lys30, Arg169, Met159, Arg18as | Asp112, Tyr134, Thr40, Leu21, Ser42, Leu13, Ile174, Gly12, Leu14, Arg11, Tyr6 |
| PSMA1-ARAFP | -849.3 | Arg157, Glu173, Arg169 | Ser14, Glu6, Lys13, | Glu23, Cys148, Met26, Met176, Ser150, Asn152, Gln146, Phe154, Met159, Asp155, Leu172, Ala158, Lys30 | Val2, Phe10, Leu11, Ala7, Ile4, Val3, Leu18, Cys1 |
| PSMA1-Core p19 | -958.3 | Thr11, Trp13, Gln5, Tyr6, Gln121, Asp155, Asn152, Ala151, Glu173 | Tyr9, Trp6, Cys2, Gln20, Arg35, Tyr31, Thr28, Lys27 | Pro15, Gln117, Arg157, Cys156, Phe154, Ser150, Cys148, Tyr153, Gly112 | Ser10, Tyr5, Met1, His17, Leu32, Tyr24, Ile21, Ile25 |
| PSMA5-RNA-directed RNA polymerase | -1029.2 | Asn13, Gly19, Gln114, Asn118, Ser159, Glu110, Glu126, Glu125, Asp127, Asp129, Gln122 | Arg503, Ser29, Asn35, Arg154, His33, Asn28, Lys20, Arg498, Arg501, Arg490 | Leu21, Phe15, Arg20, Thr161, Phe162, Ser117, Leu121, Phe123, Gly124, Ala128 | Pro495, Val499, His428, Ser431, Leu26, Ile23, His34, Mse36, Ala32, Pro496, Leu489, Leu497, Gly493 |
| PSMA5-NS4A | -741.6 | Glu233, Gln224, His227, Pro222, Asn225, Asn104 | Arg24, Thr38, Leu13, Thr40, Lys136 | Met228, Gln221, Val146, Phe226, Glu69, His73, Glu148, Thr106, Trp100, Asp71 | Leu21, Thr22, Ile18, Leu14, Ile17, Ala39, Gly12, |
| PSMA5-Envelope glycoprotein E2 | -1037.8 | Asn98, Gly131, Ala132, Arg135, Leu33, Asp9, Asp90, Thr55, Arg53 | Gly634, Thr542, Asn540, Val538, Tyr527, Arg521, Thr510, His638, Leu442, His421 | Leu58, Ile82, Lys66, Ser35, Met159, Leu81, Ala30, Ser63, Ile64, Ala83, Leu119, Thr87, Ser134, Met133, Tyr26, Val94, Lys91, Arg93, Gln97, Pro130 | ASn476, Pro567, Val636, Pro545, Val633, Ala522, Asn541, Asn548, Gly547, Leu546, Trp649, Val516, Val514, Pro525, Ala524, Gly523, Thr519, Arg424, Asn423, Ser419, Trp420, Leu413, Asn415, Asn417 |
| PSMA5-NS5A | -915.4 | Gln122, Pro17, Asn13, Phe15 | Ser81, His85, Arg41, Arg78 | Ala132, Ala128, Gly124, Phe123, Glu125, Glu126, Thr14, Leu21, Leu121, Thr161, Val24, Ser24, Ser159, Arg20, Gln25, Gly19 | Phe37, Pro35, Val34, Thr79, Phe36, Cys39, Trp84 |
| PSMA5-NS4B | -797.9 | Ser134, Ala132, Pro136, Asp84, Gln97, Thr87, Arg93, Asp90 | Gln11, Glu15, Leu6, Lys18, Arg3, Ser2, Ala1 | Met133, Phe137, Arg135, Phe101, Trp100, Ala83, Gly131, Lys86, Val94 | Leu17, Ala14, Gly10, Ile7, Leu25, Leu24, Ile21, Gln22 |
| PSMA5-Serine protease NS3 | -881.5 | Asp127, Glu126, Glu125, Leu121, Asn13, Glu25, Arg20, Pro17, Arg168, Gln164, Glu18 | Gln9, Leu13, Ala39, Arg155, His57, Gln41, Lys136, Ser133, Gly162, Arg161 | Thr14, Leu21, Phe15, Val24, Gln182, Gln178, Ala167, Tyr185, Asp166, Thr161, Gly19, Cys165, Val163 | Ala156, Gly12, Arg109, Leu14, Ile17, Leu21, Ile18, Ala157, Val132, Cys159, Tyr134, Val163 |
| PSMA5-ARAFP | -827.2 | Asp9 | Lys45 | Leu81, Ala132, Ser134, Met133, Arg135, Thr87, Phe137, Asp84, Lys91, Val94, Gln97, Asn98, Phe101, Asp90 | Thr17, Ala21, Gln8, Ala7, Leu11, Phe10, Ser14, Leu18, Phe25, Val33, Leu32, Met29, Phe109 |
| PSMA5-Core p19 | -1099.7 | Glu125, Glu126, Asp127, Phe123, Gln122, Ser117, Gln114, Arg10 | Tyr31, Tyr5, Tyr9, His17, Tyr24, Gln20, Asn19, Lys27, | Val163, Leu21, Gly11, Asn13, Ser159, Thr161, Gly160, Ala120, Phe162, Thr14, Asp9, Tyr8 | Thr28, Trp6, Phe23, Ile13, Cys2 |
| PSMB2-RNA-directed RNA polymerase | -845.3 | Asp119, Tyr120, Asn101, His99, Glu49, Gly48, Ser23, Asn24, Ile172, Phe137, Val126 | Arg498, Ser476, Arg490, Arg503, His502, Ser506, Arg505, Arg508, Lys531 | Glu2, Try134, Leu173, Ile25, Ala22, Met1 | Pro495, Gly493, Val494, Pro496, His475, Arg501, Mse423, Trp528, Leu497, Ala486, Asn483, Ile482, Val499, Ala509, Val530, Leu512 |
| PSMB2-NS4A | -756.9 | Tyr134, Glu49, Asp119 | Arg24, Leu14, Gly15, Leu13, Cys16 | Gly133, Met1, Phe171, Ile172, Arg170, Phe137, Gly48, His99, Asn101, Tyr120, Leu121 | Ile18, Thr22, Gly12, Ser20, Leu21, Thr40, Ala39, Ile17, Thr38, Ser37 |
| PSMB2-Envelope glycoprotein E2 | -1114.2 | Gly133, Ser141, Phe137, Tyr134, Arg93, Arg170, Gly48, Glu49, Arg95, Pro97, Asp52, Glu2 | Asn645, Asn623, Arg460, Met631, Arg630, Glu454, Ser450, Thr463, Glu591, Lys588, Arg587, Arg606, Glu637 | Asn101, Val47, Met1, His99, Leu92, Ala50, Phe171, Ile172, Leu38, Leu140, Ala131, His132, Tyr117, Ala125, Tyr120, Ala123, Asp119, Leu121 | Ile603, Lys628, Cys644, Cys607, Thr604, Phe627, Ser499, Tyr624, Met456, Cys459, Tyr632, Ile629, Pro453, Ala457, Phe586, Asp584, Pro461 |
| PSMB2-NS5A | -1019.1 | Tyr134, Glu2, Arg170, Asn101, Tyr120, Asn24, His99, Glu49, Gly48 | Arg78, Thr79, Arg73, Gly33, Pro35, Asp154, Asn82, Gln40 | Tyr117, His132, Ala131, Ala136, Phe137, Gly133, Phe171, Leu173, Ile172, Val47, Met1, Asp119, Leu121, Ala50, Ala22 | Trp84, Pro77, Leu32, Val34, Phe36, Ser81, Cys39, Arg41, His85, Phe37 |
| PSMB2-NS4B | -839.0 | Asp144, Tyr117, Tyr120, His99, Glu49, Asp52, Gly48, Glu2 | Ser2, Arg3, Gln11, Ala14, Lys20 | Tyr134, Gly133, His132, Phe137, Phe129, Ala127, Lys126, Ala136, Ala131, Leu140, Asp119, Pro128, Leu121, Asn101, Met1 | Ala1, Leu6, Ile7, Leu24, Ile7, Leu6, Ala1 |
| PSMB2-Serine protease NS3 | -905.7 | Asn24, Ile25, Tyr134, Gly48, Glu49, Tyr120, Asp119, Tyr117, Arg93 | Gln9, Ser42, Lys136, Arg109, Arg130, Val163, Lys165, Arg16, Ala157, Ser133 | Glu2, Met1, Ile172, Leu173, Ala22, Ser23, His99, Leu124, Phe137, Ala136, His132, Asn101, Leu92, Leu121, | Thr40, Gln41, His110, His57, Gly137, Gly58, Leu13, Tyr134, Gly162, Cys159, Val158, Ala156, Phe154, Leu135, Val132, Ser139, Pro131 |
| PSMB2-ARAFP | -943.2 | Arg170, Tyr134, Arg93, Arg86 | Phe109, Ser108, Asp28, Thr15, Glu6, Val3 | Tyr120, Leu121, His99, pro97, Leu92, Ser94, Lys169, Ile72, Phe171, Leu138, Phe137, Ala89, Asp90 | Ile37, Leu32, Ser14, Leu18, Phe10, Leu11, Ile4, Ala7, Val33, Met29, Phe25, Ala21 |
| PSMB2-Core p19 | -1056.7 | Asn24, Tyr117, Asp144, Gly133, Asp119, Tyr120, Ile25 | Lys27, Thr12, Tyr5, Tyr9, Lys3, Trp6, Asn19 | Leu173, Ile172, Tyr134, Met1, Ala22, Phe137, Ala136, Ala131, His132, Asn101, Leu121, Glu49 | Phe23, Leu16, Asn8, Ser10, Gln20, Ile13, Tyr14, Ile21, Ile25, Thr28, Tyr24 |
